# Calciprotein particle-induced cytotoxicity via lysosomal dysfunction and altered cholesterol distribution in renal epithelial HK-2 cells

**DOI:** 10.1038/s41598-020-77308-3

**Published:** 2020-11-18

**Authors:** Rina Kunishige, Mai Mizoguchi, Asako Tsubouchi, Kenjiro Hanaoka, Yutaka Miura, Hiroshi Kurosu, Yasuteru Urano, Makoto Kuro-o, Masayuki Murata

**Affiliations:** 1 Department of Life Sciences, Graduate School of Arts and Sciences, The University of Tokyo, 3-8-1 Komaba, Meguro-ku, Tokyo, 153-8902 Japan; 2 Graduate School of Pharmaceutical Sciences, The University of Tokyo, 7-3-1 Hongo, Bunkyo-ku, Tokyo, 113-0033 Japan; 3 Division of Anti-Aging Medicine, Center for Molecular Medicine, Jichi Medical University, 3311-1 Yakushiji, Shimotsuke, Tochigi 329-0498 Japan; 4 Graduate School of Medicine, The University of Tokyo, 7-3-1 Hongo, Bunkyo-ku, Tokyo, 113-0033 Japan; 5 CREST (Japan) Agency for Medical Research and Development (AMED), 1-7-1 Otemachi, Chiyoda-ku, Tokyo, 100-0004 Japan; 6 Cell Biology Center, Institute of Innovative Research, Tokyo Institute of Technology, 4259 Nagatsuta, Midori-ku, Yokohama, Kanagawa 226-8503 Japan

**Keywords:** Autophagy, Lysosomes, Mechanisms of disease, Sensors and probes

## Abstract

Dietary phosphate overload induces chronic kidney disease (CKD), and calciprotein particles (CPPs), a form of nanoparticle comprising calcium phosphate and serum proteins, has been proposed to cause renal toxicity. However, the mechanism of CPP cytotoxicity in renal tubular cells is unknown. Here we show that in renal proximal tubular epithelial HK-2 cells, endocytosed CPPs accumulate in late endosomes/lysosomes (LELs) and increase their luminal pH by ~ 1.0 unit. This results in a decrease in lysosomal hydrolase activity and autophagic flux blockage without lysosomal rupture and reactive oxygen species generation. CPP treatment led to vulnerability to H_2_O_2_-induced oxidative stress and plasma membrane injury, probably because of autophagic flux blockage and decreased plasma membrane cholesterol, respectively. CPP-induced disruption of lysosomal homeostasis, autophagy flux and plasma membrane integrity might trigger a vicious cycle, leading to progressive nephron loss.

## Introduction

Chronic kidney disease (CKD) is a serious health problem worldwide because of its rapidly increasing prevalence and heavy economic burden^1^. Dietary phosphate overload induces CKD in healthy animals^2^ and individuals^3^. An increase in phosphate excretion per nephron induces renal injury in rats, and bisphosphonate administration, which inhibits the formation of calcium phosphate (CaPi) crystals, alleviates this injury^4,5^. Therefore, an increase in phosphate excretion per nephron can raise the phosphate concentration in renal tubular fluid and trigger precipitation of CaPi crystals, which can cause renal tubular damage^6^. Once the number of functional nephron is decreased, phosphate excretion per nephron must increase to maintain the phosphate balance, further promoting CaPi precipitation, leading to CKD progression^6^. Because hyperphosphatemia observed in patients with end-stage renal disease is a major risk factor for cardiovascular events and mortality, researchers have focused on CaPi crystals present in the blood, which may exert cytotoxic effects on vascular smooth muscle cells. In general, high serum phosphate levels are observed only in the late stages of CKD and not in early-stage, where serum phosphate balance is maintained by an enhanced fractional excretion of phosphate per functioning nephron. Indeed, a micropuncture study conducted by Bank et al. directly measured phosphate concentration of the renal tubular fluid in subtotal-nephrectomised rats showing normal phosphorus level in the serum but higher in the tubular fluid. This compensatory mechanism, in contrast, might lead to CaPi crystal formation in the primary urine. Thus, we focused on the potential role of CaPi crystals in the urine, which may exert deleterious effects from the onset of CKD.

CaPi has potential as a nanomaterial in biomedical implants, DNA delivery, and drug delivery^7^. Many in vitro experiments have shown that CaPi nanoparticles have significant cytotoxicity, and it is necessary to understand the biologic effects of CaPi. Fast dissolution of CaPi under acidic conditions of lysosomes induces cell necrosis with lysosomal membrane permeabilization (LMP)^7^. Studies have also reported endosome damage–induced autophagy^8^ and autophagy flux perturbation^9^. Therefore, endogenous CaPi particles formed in the proximal tubular lumen might affect the autophagy–lysosomal pathway.

Renal proximal tubular epithelial cells (HK-2 cells) depend on aerobic respiration in mitochondria to fulfil the high energy demand for active transport of electrolytes, and therefore, autophagy-mediated turnover of worn-out mitochondria is essential^10,11^. Autophagy blockage leads to accumulation of damaged mitochondria generating excess reactive oxygen species (ROS) that activates the nucleotide-binding domain-like receptor protein 3 (NLRP3) inflammasome^12^. Therefore, autophagy dysfunction might contribute to CKD progression via inflammation and fibrosis^13^. Indeed, long-term autophagy deficiency exaggerates mitochondrial dysfunction in mice, accelerating kidney fibrosis and dysfunction^14^. Autophagy also protects cells from dysfunction by digesting damaged proteins and organelles after ischaemia–reperfusion injury (IRI) and treatments with nephrotoxins^15^. In autophagy-deficient mice, IRI increases proximal tubular cell death with p62 accumulation^16^. Therefore, autophagy dysfunction might promote CKD by making cells susceptible to stress, leading to functional nephron loss.

In the plasma, fetuin-A, a serum protein, sequesters CaPi particles and inhibits crystal growth and aggregation, preventing CaPi precipitation in extraosseous tissues. Fetuin-A-deficient mice suffer from severe ectopic calcification of various organs^17^. In vitro, fetuin-A decreases the detrimental effects of CaPi in vascular smooth muscle cells and macrophages^18,19^. Calciprotein particles (CPPs) (i.e. CaPi bound to serum proteins, mainly fetuin-A) reflect the actual state of extraosseous CaPi in vivo. Fetuin-A is present in the proximal tubular lumen^20^, and CaPi precipitates may bind to fetuin-A and form CPPs; these precipitates may have cytotoxic effects different from inorganic CaPi, when they accumulate in pathological conditions^21^.

Although the effect of inorganic CaPi crystals has been reported on renal epithelial cells, no studies have reported the effects of CPPs on cytotoxicity. In this study, we synthesized CPPs in vitro by incubating inorganic CaPi particles in a serum-containing medium to better mimic CaPi particles, which are expected to exist in the renal tubular fluid, and assessed the detrimental effects of CPPs on HK-2 cells. CPP-induced disruption of lysosomal homeostasis, autophagy flux, and plasma membrane integrity might trigger a vicious cycle, leading to progressive renal tubular damage.

## Results

### Caveola-dependent endocytosis and macropinocytosis induce CPP internalization in HK-2 cells

**Figure 1:**
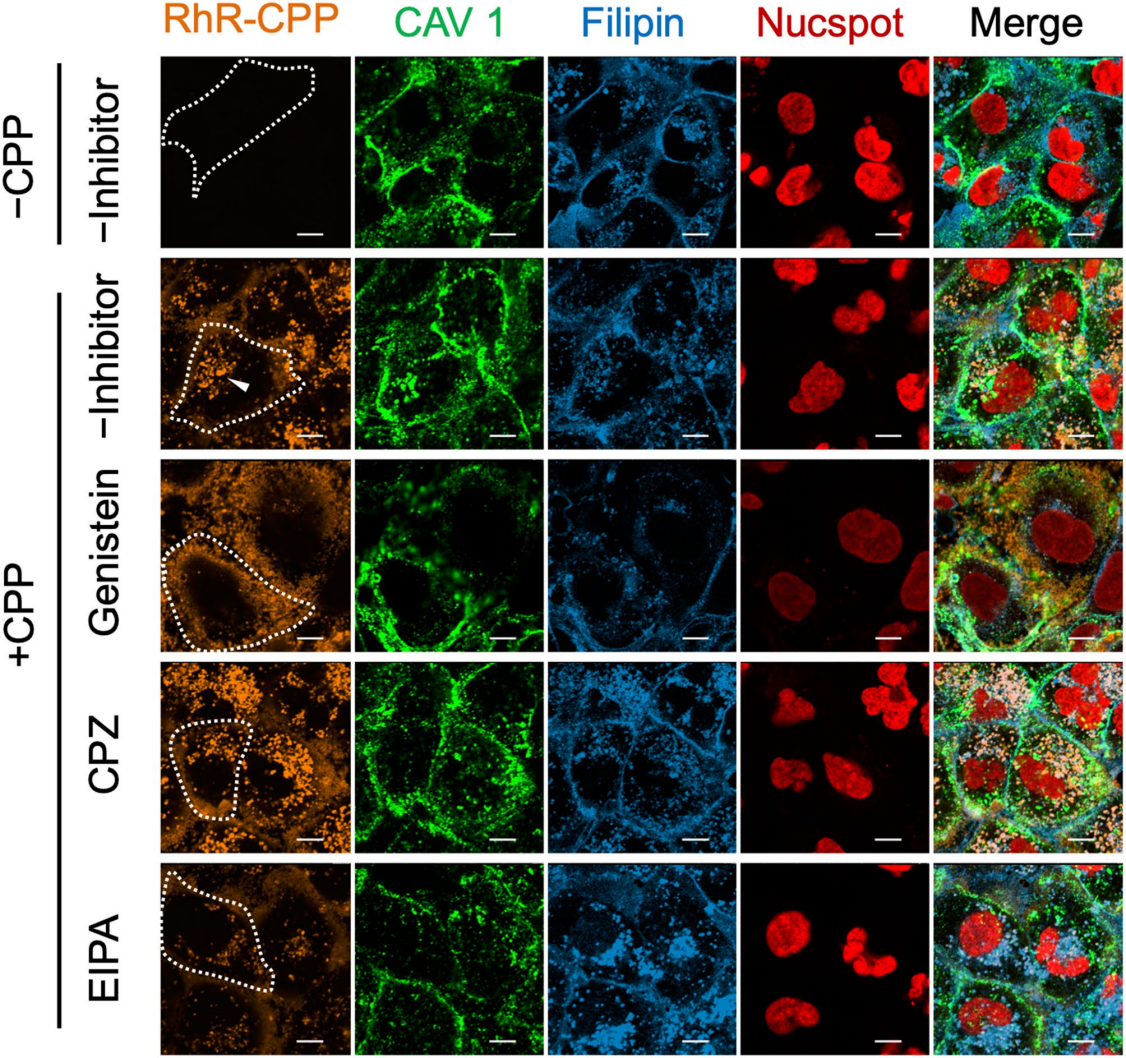
Caveola-dependent endocytosis and macropinocytosis induce CPP internalization in HK-2 cells. Confocal microscopy images of cellular uptake of RhR-CPPs in HK-2 cells. Cells were incubated with RhR-CPPs for 12 h, fixed, Chol was labelled with filipin, and plasma membrane was labelled by Cav1 antibody. For endocytosis-inhibiting conditions, we pre-treated HK-2 cells with each inhibitor (200 μM caveola-dependent endocytosis inhibitor genistein, 15 μM clathrin endocytosis inhibitor CPZ or 25 μM macropinocytosis inhibitor EIPA) for 30 min and further incubated them with RhR-CPPs and the inhibitors for 12 h. Arrowheads point to RhR-CPPs incorporated into HK-2 cells. The fluorescence seen along the cell outline (white dotted line) is RhR-CPPs bound to the cell membrane. Genistein significantly decreased and CPZ increased RhR-CPP internalization. EIPA decreased CPP binding to the cell membrane and RhR-CPP internalization. Scale bar = 10 μm. RhR-CPPs, 5(6)-RhR-dRIS-labelled calciprotein particles; Cav1, caveolin-1; CPZ, chlorpromazine.

HK-2 cells were incubated with a medium containing fluorescence 5(6)-RhR-dRIS-labelled CPPs (RhR-CPPs) for various time periods, fixed, and then observed using a confocal microscope. RhR-CPPs attached to the cell surface gradually endocytosed and finally accumulated in peri-nuclear vesicles in HK-2 cells within 24 h of incubation (Fig. 1); some RhR-CPPs remained attached to the cell surface.

In control samples without any inhibitor, endocytic vesicles containing RhR-CPPs accumulated near the nucleus after 24 h of incubation (Fig. 1, + CPP, − Inhibitor). In contrast, genistein, a caveola-dependent endocytosis inhibitor, significantly inhibited the peri-nuclear accumulation of RhR-CPPs, while more RhR-CPPs attached to the cell surface (Fig. 1, Genistein). Dual fluorescence imaging using HK-2 cells transiently expressing caveolin-1 fused with green fluorescent protein (GFP) (Cav1-GFP) showed that RhR-CPPs accumulated in Cav1-GFP-positive membrane structures (Supplementary Fig. S1). The macropinocytosis inhibitor 5-(*N*-ethyl-*N*-isopropyl)-amiloride (EIPA) decreased both RhR-CPP attachment to the cell surface and RhR-CPP internalization in HK-2 cells (Fig. 1, EIPA). In contrast, chlorpromazine, a clathrin-dependent endocytosis inhibitor, increased RhR-CPP intracellular accumulation (Fig. 1, CPZ). Therefore, CPPs are internalized mainly through caveola-dependent endocytosis and macropinocytosis.

### CPPs affect cholesterol intracellular transport and localization, and increase vulnerability towards plasma membrane damage

**Figure 2:**
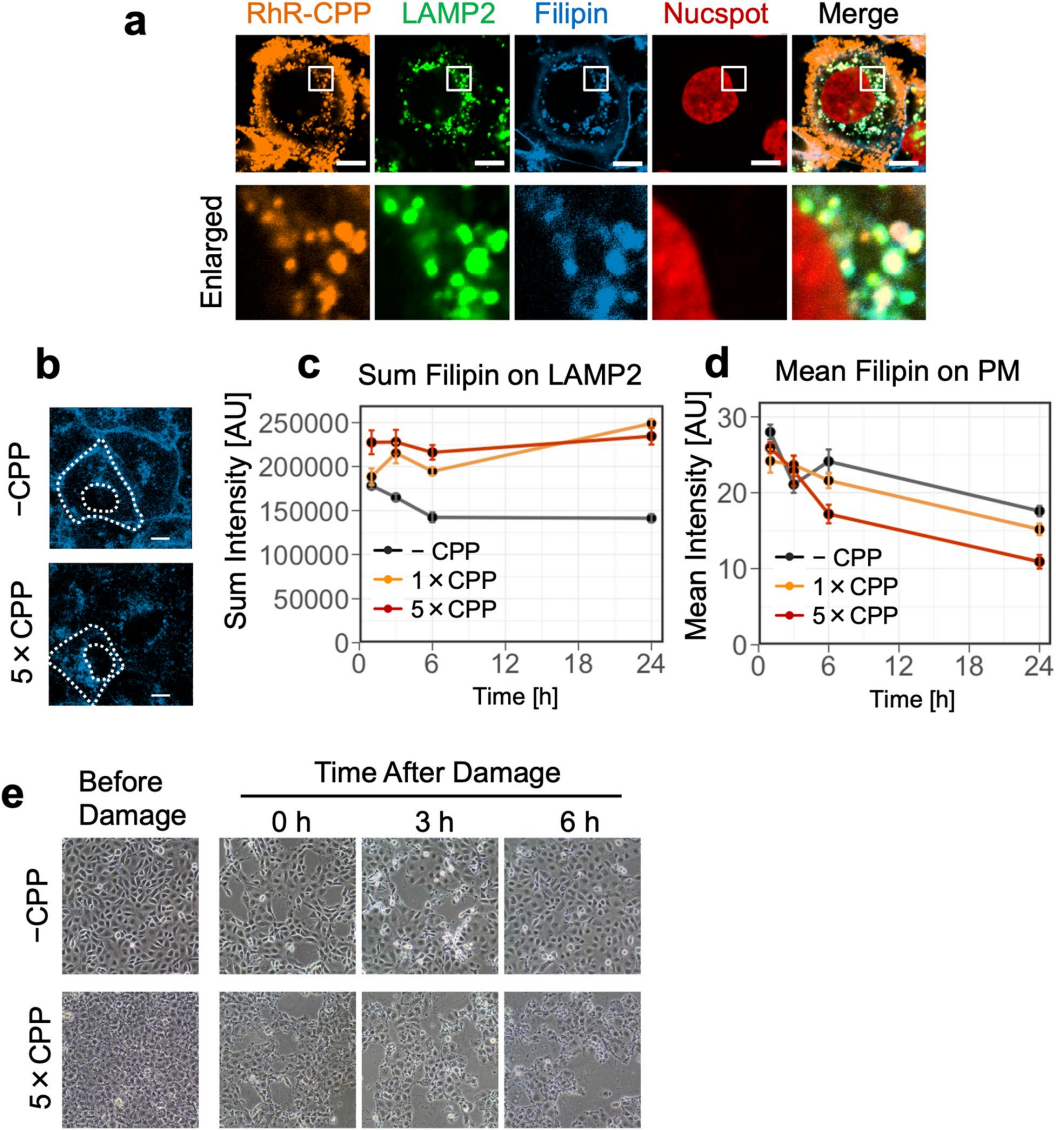
CPPs affect intracellular localization of Chol, leading to vulnerability towards plasma membrane injury. (**a**) Confocal microscopy images showing co-localization of RhR-CPPs and Chol in LELs. RhR-CPPs were uptaken by HK-2 cells for 3 h and fixed in 4% PFA. Chol was labelled by filipin and LELs by LAMP2 antibody. Lower panels are a magnified image of the white boxes in upper panels. Scale bar = 10 μm. (**b**) Filipin-labelled Chol of HK-2 cells incubated with or without CPPs for 24 h. CPPs decreased plasma membrane Chol and increased intracellular Chol. White dotted lines outline the cell and the nucleus. Scale bar = 10 μm. (**c**) Graph showing increased LEL Chol in HK-2 cells incubated with CPPs. For microscopic image quantification, we created an LEL mask from the LAMP2-positive region (Supplementary Fig. S3). We calculated the sum intensity of filipin fluorescence on the LAMP2-positive region/cell. Data represent results from four wells (*n* = 4), expressed as mean ± SEM. (**d**) Graph showing decreased plasma membrane Chol in HK-2 cells incubated with CPPs. For microscopic image quantification, we created a plasma membrane mask from Cav1- and ZO-1-positive regions (Supplementary Fig. S3). We calculated the mean intensity of filipin fluorescence on the plasma membrane region/cell. Data represent results from four wells (*n* = 4), expressed as mean ± SEM. (**e**) Microscopic image showing that CPP-treated HK-2 cells are vulnerable to plasma membrane injury. We incubated HK-2 cells with or without CPPs for 12 h and subjected them to plasma membrane damage using 425–600 μm glass beads. CPP-untreated HK-2 cells showed gradual recovery of the disrupted cell monolayer, while we observed significant cell death in CPP-treated HK-2 cells. PM, plasma membrane; Cav1, caveolin-1; RhR-CPPs, 5(6)-RhR-dRIS-labelled calciprotein particles; LELs, late endosomes/lysosomes.

To characterize the RhR-CPP-loaded endocytic vesicles, immunofluorescence analysis was performed using antibodies against various endocytic or organelle markers. RhR-CPP-treated endocytic vesicles accumulating in the peri-nuclear region were lysosomal-associated membrane protein 2 (LAMP2)-positive vesicles, indicating that they are late endosomes/lysosomes (LELs) (Fig. 2a). More filipin-labelled cholesterol (Chol) was concentrated in the peri-nuclear region in CPP-treated HK-2 cells (Fig. 2b), presumably RhR-CPP-containing LELs (Fig. 2a). A biochemical experiment indicated affinity of CPP towards Chol (Supplementary Fig. S2a), and image-based quantitative microscopic analysis (Supplementary Fig. S3) showed alteration in the amount of filipin-labelled Chol in LAMP2-positive-LELs and plasma membrane. The amount of Chol in LELs increased over time, which was associated with reciprocal decrease in plasma membrane Chol during the course of 6 h incubation of HK-2 cells with CPPs (Fig. 2b–d and Supplementary Fig. S4).

TopFluor cholesterol (TF-Chol) in the plasma membrane was internalized and then gradually exported from CPP-untreated HK-2 cells (Supplementary Fig. S5a). In contrast, CPP-treated HK-2 cells showed a slight delay in TF-Chol efflux (Supplementary Fig. S5b,c), and more TF-Chol accumulated in peri-nuclear vesicles (Supplementary Fig. S5d). Therefore, because of their binding affinity towards Chol, CPPs might bind to plasma membrane Chol and together internalize into the LELs and may also interrupt Chol trafficking from LELs to the plasma membrane, decreasing plasma membrane Chol.

Cholesterol is known to be involved in plasma membrane repair following damage^22^. Indeed, plasma membrane Chol depletion by methyl-β-cyclodextrin (MβCD) increased the cell injury probability following wounding by glass beads^23^ (Supplementary Fig. S6). To examine the effect of plasma membrane Chol depletion by CPP treatment, the plasma membrane of HK-2 cells was wounded by glass beads after a 12-h incubation with or without CPP. In CPP-untreated HK-2 cells, the disrupted cell monolayer mostly recovered within 6 h of incubation. In contrast, recovery from the injury was significantly delayed in CPP-treated HK-2 cells (Fig. 2e), indicating that plasma membrane Chol depletion by CPPs induces vulnerability of the plasma membrane integrity to mechanical damage^22^.

### Cellular uptake of CPPs increases luminal pH of LELs and causes lysosomal swelling

**Figure 3:**
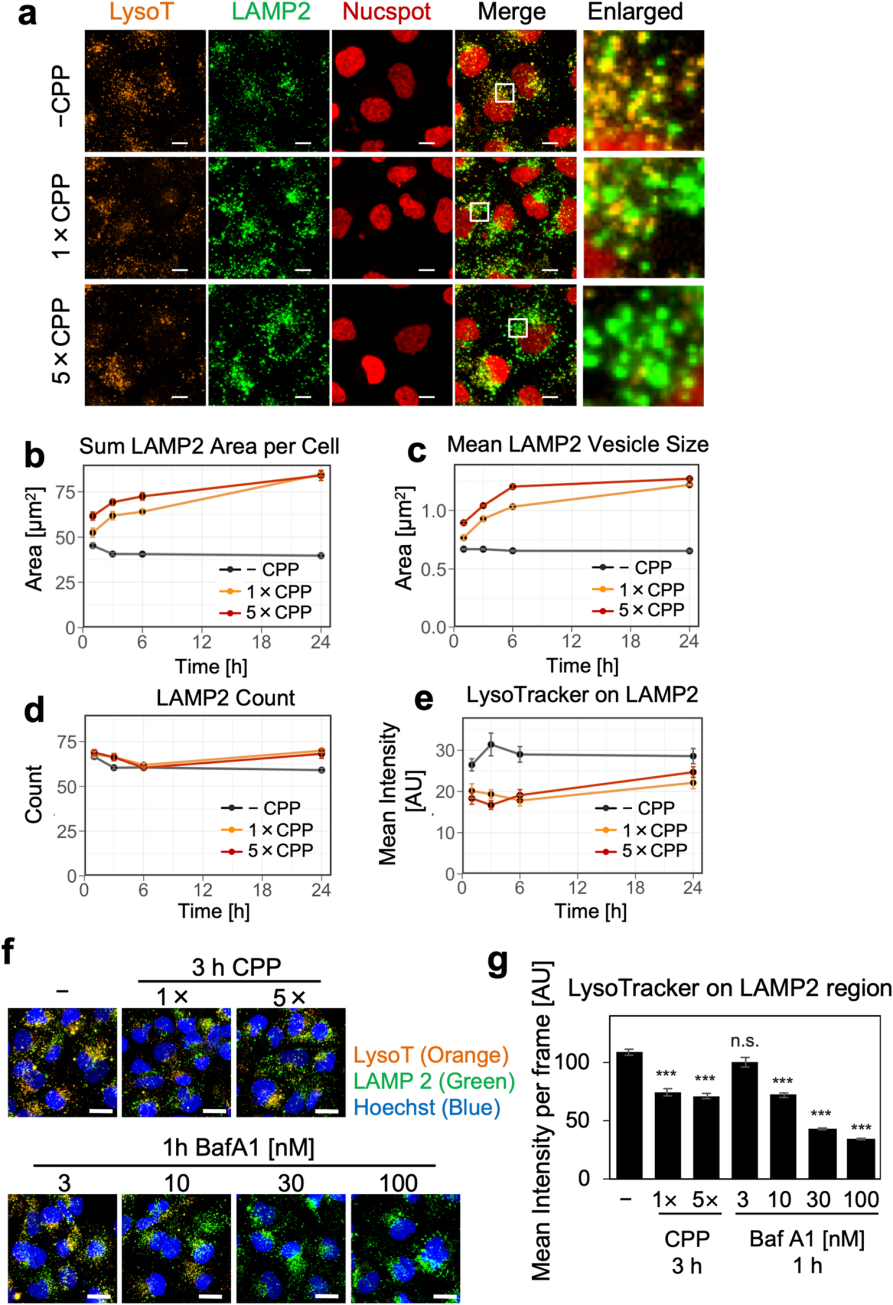
CPPs cause lysosomal swelling and increase luminal pH of LELs. (**a**) Confocal microscopy images showing decrease of LysoTracker staining, indicating increased luminal pH and lysosomal swelling in HK-2 cells incubated with CPPs. We stained HK-2 cells incubated for 1, 3, 6 and 24 h in low (1×) or high (5×) CPP concentrations with LysoTracker and fixed in 4% PFA. We performed immunofluorescence using LAMP2 antibody in order to define the LEL region. Panels show representative images of HK-2 cells incubated with or without CPPs for 24 h. Scale bar = 10 μm. (**b**) Temporal change in the sum LAMP2 area/cell (μm^2^) after incubation with or without CPPs. Addition of CPPs increased the LAMP2 region area. Data represent results from four wells (*n* = 4), expressed as mean ± SEM. (**c**) Temporal change in mean LAMP2 vesicle size/cell (μm^2^) after incubation with or without CPPs. Addition of CPPs increased the LAMP2 vesicle size (*n* = 4), expressed as mean ± SEM. (**d**) Temporal change in the number of LAMP2 vesicles/cell. Addition of CPPs slightly increased LAMP2 vesicles/cell after 24 h (*n* = 4), expressed as mean ± SEM. (**e**) Temporal change in mean fluorescence of LysoTracker on the LAMP2 region/cell after incubation with or without CPPs. Addition of CPPs decreased the LysoTracker intensity on the LAMP2 region (*n* = 4), expressed as mean ± SEM. (**f**) Comparison of the LEL alkalizing effect between CPP and BafA1. We treated HK-2 cells with CPPs or BafA1 for each hour, followed by LysoTracker staining. After PFA fixation, we performed immunofluorescence using LAMP2 antibody. Scale bar = 20 μm. (**g**) Quantification of images obtained in (**f**). The bar graph shows the mean fluorescence intensity of LysoTracker on the LAMP2 region. In CPP-treated HK-2 cells, the decrease in LysoTracker staining after 3 h, indicating a luminal pH increase, was comparable to that of 10 nM BafA1 treatment for 1 h. Data represent results from eight frames (*n* = 8), expressed as mean ± SEM. ***P < 0.001, *ns* not significant. *BafA1* bafilomycin A1.

Endocytosed nanoparticles such as silica particles and uric acid crystals damage lysosomes and cause cytotoxicity^24–26^. Therefore, we examined whether CPP had similar effects on lysosomes by investigating changes in pH, size, or number of lysosomes in each cell using image-based quantitative microscopic analysis. Immunofluorescence analysis showed that the LAMP2-positive area/cell (μm^2^) gradually increased 1–6 h after CPP treatment of HK-2 cells (Fig. 3a,b and Supplementary Fig. S7d). This increase was caused by an enlargement of individual LELs (Fig. 3a,c and Supplementary Fig. S7e), because the number of LAMP2-positive LELs/cell remained constant, only showing a slight increase after 24 h (Fig. 3d and Supplementary Fig. S7f.). Therefore, LELs in CPP-treated HK-2 cells did not significantly increase in number but rather swelled in size compared with CPP-untreated HK-2 cells.

In addition, we observed that CPP treatment reduced the fluorescence intensity of LysoTracker in LAMP2-positive LELs, indicating an increase in luminal pH (Fig. 3a and Supplementary Fig. S7b). Quantification of the mean fluorescence intensity of LysoTracker in LAMP2-positive regions indicated a rapid luminal pH increase in LELs within 1 h after CPP treatment, which persisted through 24 h of incubation (Fig. 3e and Supplementary Fig. S7g). HK-2 cells incubated with 10 nM bafilomycin A1 (BafA1), which increases the luminal pH by inhibiting vacuolar H^+^-ATPase (v-ATPase) on lysosomal membranes, for 1 h showed a comparable pH increase in CPP-treated HK-2 cells for 3 h (Fig. 3f,g).

Inhibition of endosomal acidification by BafA1 is known to disturb transferrin recycling^27^. However, transferrin recycling was not affected by CPP treatment (Supplementary Fig. S8). Therefore, CPPs likely increase the luminal pH of LELs, although not to the extent of inhibiting transferrin recycling.

### Luminal pH of LELs increases by 1.0 unit in CPP-treated HK-2 cells

SiRpH 5.5-dextran (SiRpH 5.5-Dex) (p*K*_a_ = 5.5) and SiRpH 5-Dex (p*K*_a_ = 6.1) are novel ratiometric, pH-sensitive fluorescent probes that enable more accurate pH measurement compared with single-wavelength probes, such as LysoTracker^28^ (Supplementary Fig. S9a,b). As the size of LAMP2-positive vesicles changes, a decrease in the mean fluorescence intensity of LysoTracker may not necessarily reflect an increase in pH. In addition, another advantage of Dex-conjugated probes is that they can specifically label endosomes and lysosomes, whereas LysoTracker is membrane-permeable and non-specifically labels the acidic compartment of cells.

**Figure 4:**
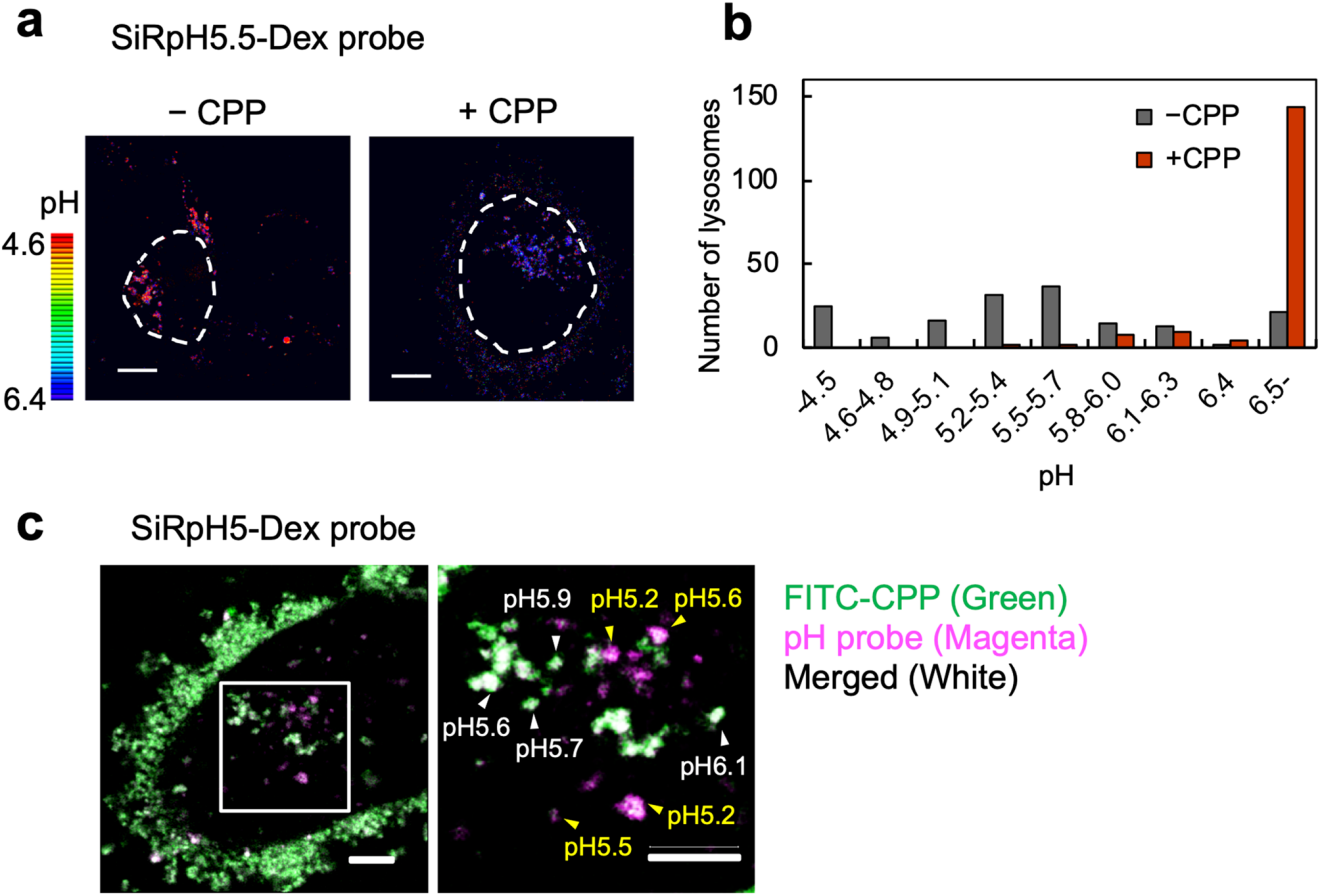
A novel ratiometric probe revealed increased luminal pH of LELs by 1.0 unit in CPP-treated HK-2 cells. **(a)** Fluorescence ratio images showing increased luminal pH of LELs in CPP-treated HK-2 cells. We incubated HK-2 cells with SiRpH5.5-Dex for 6 h in the presence or absence of CPPs. White dotted lines outline the cell. Scale bar = 10 μm. **(b)** Histogram showing quantified pH of > 160 LELs under each condition in **(a)**. We calculated the pH of individual LELs using the calibration curve (Supplementary Fig. S9c). CPP incubation increased the mean pH from 5.48 to 6.44. The luminal pH of some LELs, especially with CPPs, exceeded the measurable range of the SiRpH 5.5-Dex probe (~ 6.5) and was calculated as 6.5. **(c)** Microscopic image showing increased luminal pH in LELs containing CPPs. We incubated HK-2 cells with SiRpH5-Dex and FITC-CPP for 6 h. Green indicates FITC-CPP, and magenta indicates probe fluorescence. We calculated the pH of individual LELs using the calibration curve (Supplementary Fig. S9g). Yellow and white arrowheads indicate LELs without and with FITC-CPP, respectively. Although vesicles with minimum fluorescence intensity of FITC-CPP were regarded as lacking CPP, they may still contain undetectable amounts of CPPs. Scale bar = 10 μm. FITC-CPP, fluorescein isothiocyanate–labelled CPP.

We incubated HK-2 cells with or without CPP for 6 h in a medium containing the SiRpH 5.5-Dex probe, enabling their transport to lysosomes. After live imaging, we fixed the cells and conducted in situ pH calibration by treating the cells with sodium phosphate (NaPi) buffer ranging in pH from 3 to 6.5 (Supplementary Figs. S9c,d). Finally, we measured the pH of > 160 LELs under conditions with and without CPPs (Fig. 4a). The mean pH of LELs increased by ~ 1.0 unit after CPP treatment; the mean pH with and without CPPs was 6.44 and 5.48, respectively (Fig. 4a,b).

The alkalization effect of 1 h treatment with 10 nM BafA1 was equivalent to that of CPPs (Fig. 3g), which was ~ 1.0 unit increase in pH from 5.14 to 6.11 (Supplementary Fig. S9e,f). These results indicated that both LysoTracker and SiRpH 5.5-Dex methods were reliable and that CPPs increased the luminal pH of LELs.

We also confirmed that the luminal pH increase was due to CPP uptake. As shown in Fig. 4c, the pH was higher in vesicles containing fluorescein isothiocyanate–labelled CPP (FITC-CPP) compared with those without FITC-CPP in the same cell. Therefore, endocytosed CPPs increase the pH of CPP-containing LELs by ~ 1.0 unit in HK-2 cells.

### CPPs do not induce lysosomal membrane permeabilization or ROS generation

**Figure 5:**
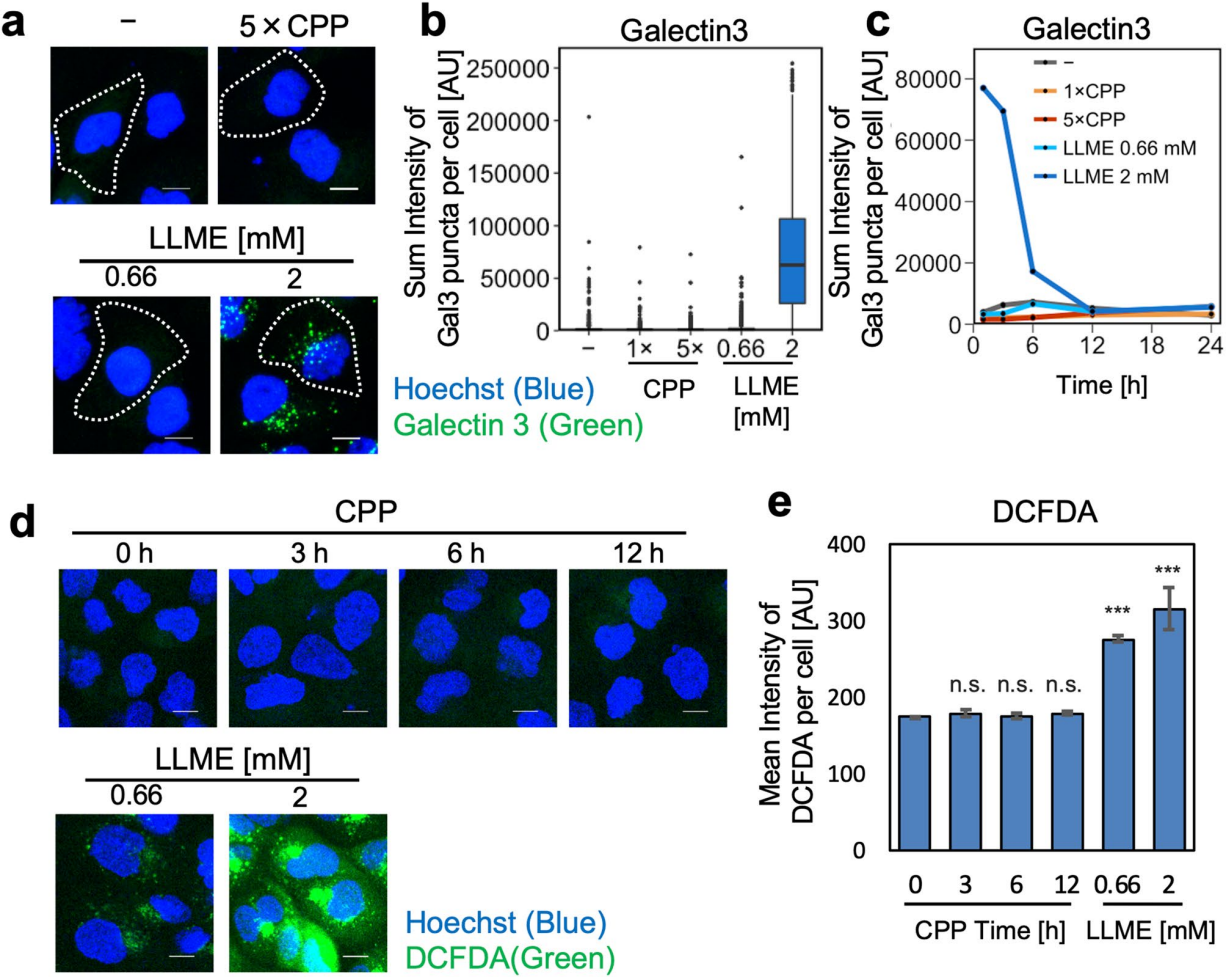
CPPs do not induce lysosomal membrane permeabilization or ROS generation. (**a)** Microscopic image showing induction of lysosomal membrane permeabilization by 2 mM LLME but not CPP. We treated HK-2 cells under each condition for 1 h, fixed them in 4% PFA, and performed immunofluorescence using galectin-3 antibody. Scale bar = 10 μm. **(b)** Quantification of images obtained in (**a**). Box plots show the sum fluorescence intensity of galectin-3 puncta/cell. **(c)** Line graph representing temporal change in the sum intensity of galectin-3 puncta/cell, incubated with or without CPPs or LLME. CPPs did not induce lysosomal membrane permeabilization at any time point. **(d)** ROS detection using the DCFDA/H2DCFDA kit performed with HK-2 cells treated under each condition. We observed ROS generation only after LLME treatment, not after CPP treatment. (**e)** Quantification of images obtained in (**d**). Data represent results from three frames (*n* = 3), expressed as mean ± SEM. ***P < 0.001, n.s: not significant. LLME, L-leucyl-L-leucine methyl ester; DCFDA, 2′,7′-dichlorofluorescin diacetate.

Endocytosed nanoparticles such as silica particles and urate crystals induce lysosomal membrane permeabilization, increasing the luminal pH and ROS generation^25,29,30^. Thus, we asked whether the CPP-induced increase of luminal pH was due to lysosomal membrane permeabilization. We observed bright fluorescent spots of condensed galectin-3, an lysosomal membrane permeabilization marker, in HK-2 cells treated with 2 μM L-leucyl-L-leucine methyl ester (LLME) that induces lysosomal membrane permeabilization. In contrast, there were no fluorescent spots in CPP-treated HK-2 cells at any time point (Fig. 5a–c). In addition, the 2′,7′-dichlorofluorescin diacetate (DCFDA) assay showed ROS generation in HK-2 cells treated with LLME for 4 h but no ROS generation in HK-2 cells treated with CPPs for 1, 12 and 24 h (Fig. 5d,e). These results indicated endocytosed CPPs in HK-2 cells do not cause lysosomal membrane permeabilization or ROS generation.

### CPPs induce TFEB-/TFE3-mediated lysosomal stress response

Transcription factors EB and E3 (TFEB and TFE3) are phosphorylated by mammalian target of rapamycin complex 1 (mTORC1) and sequestered in the cytoplasm under unstressed conditions^31^. Under lysosomal stress conditions, such as increased luminal pH, mTORC1 dissociates from the lysosomal surface, a major locus for the activation of mTORC1, resulting in mTORC1 inactivation^32^. Dephosphorylated TFEB/TFE3 translocate to the nucleus and induce the expression of the autophagy- and lysosome-related genes, thereby alleviating lysosomal stress conditions^31^. Thus, we examined whether these events were also observed under increased pH conditions of LELs induced by CPP.

**Figure 6:**
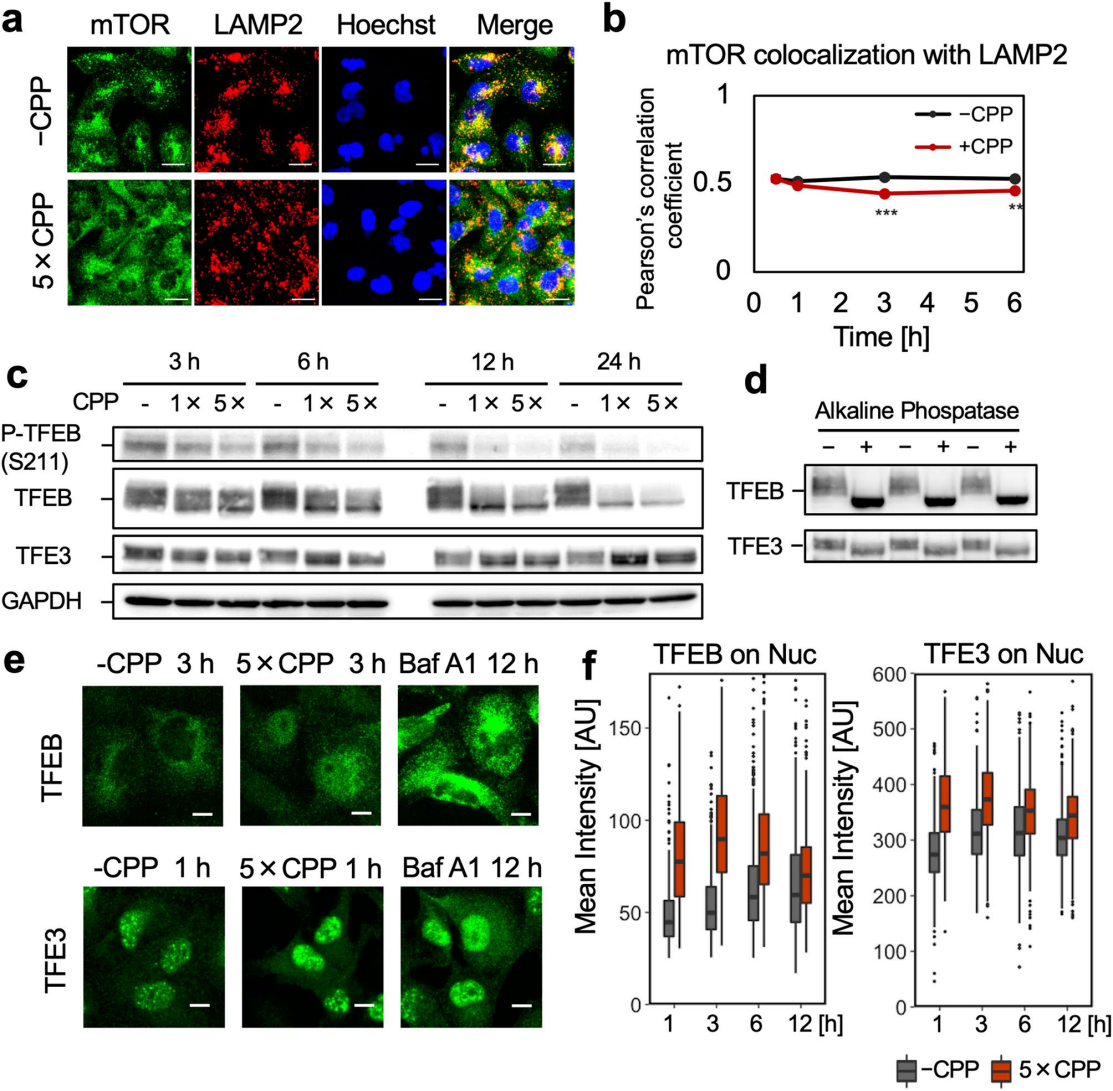
CPPs induced mTORC1 dissociation from lysosomes and trigger TFEB-/TFE3-mediated lysosomal stress response. **(a)** Immunofluorescence of mTOR and LAMP2 in HK-2 cells incubated with or without CPPs for 3 h. The merge image shows decreased co-localization between mTOR and LAMP2, indicating mTORC1 dissociation from the lysosome surface. Scale bar = 20 μm. (**b)** Line graph showing decreased co-localization of mTOR and LAMP2 in HK-2 cells incubated with CPP. Data represent results from six frames (*n* = 6), expressed as mean ± SEM. **P < 0.01, ***P < 0.001. **(c)** WB showing CPP-induced dephosphorylation of TFEB (S211) and a slight band shift towards a lower molecular weight in TFEB and TFE3. HK-2 cells incubated for 3, 6 and 24 h in low (1×) or high (5×) CPP concentrations was analysed. Full-length blots are presented in Supplementary Fig. S16a. **(d)** WB showing slight band shift in TFEB/TFE3 towards a lower molecular weight in the dephosphorylated lysate. HK-2 cell lysate treated with alkaline phosphatase was analysed. Full-length blots are presented in Supplementary Fig. S16B. **(e)** Immunofluorescence examination of nuclear translocation of TFEB and TFE3. TFEB showed nuclear translocation in CPP- and BafA1-treated HK-2 cells. The fluorescent intensity of nuclear TFE3 increased in CPP- and BafA1-treated HK-2 cells. Scale bar = 10 μm. **(f)** Box plot showing the mean fluorescence intensity of TFEB/TFE3 in the nucleus. The nuclear intensity of TFEB/TFE3 increased, especially at 1–3 h after CPP addition.

As shown in Fig. 6a,b, mTOR significantly dissociated from LAMP2-positive LELs after CPP treatment, indicating that CPPs induce mTORC1 dissociation from the lysosomal surface, leading to mTORC1 inactivation. In the western blotting (WB) analysis of TFEB and TFE3 (Fig. 6c), we observed a minor band shift towards a lower molecular weight for both TFEB and TFE3 in CPP-treated HK-2 cells. This band shift represented dephosphorylation, as HK-2 cell lysate treated with alkaline phosphatase (ALP) showed a similar band shift (Fig. 6d) and WB using anti-phosphorylated TFEB (pSer211) antibody directly confirmed reduction of phosphorylated TFEB (Fig. 6c).

Immunofluorescence analysis showed prominent translocation of TFEB from the cytosol to the nucleus, and TFE3 also appeared to increase in the nucleus (Fig. 6e). Quantification of TFEB and TFE3 fluorescence intensities in the nucleus showed a rapid increase starting from 1 to 12 h after CPP treatment (Fig. 6f) and also BafA1 treatment (Supplementary Fig. S10a). In addition, quantitative polymerase chain reaction (qPCR) assay showed a significant increase in the expression of several TFEB/TFE3 target genes, such as *CTSD*, *MCOLN1* and *SQSTM1* (Supplementary Fig. S10b). These results showed that CPPs induced dephosphorylation and translocation of TFEB and TFE3, followed by lysosomal stress–responsive gene expression under increased pH conditions in LELs.

### CPPs decrease hydrolase activity in lysosomes

**Figure 7:**
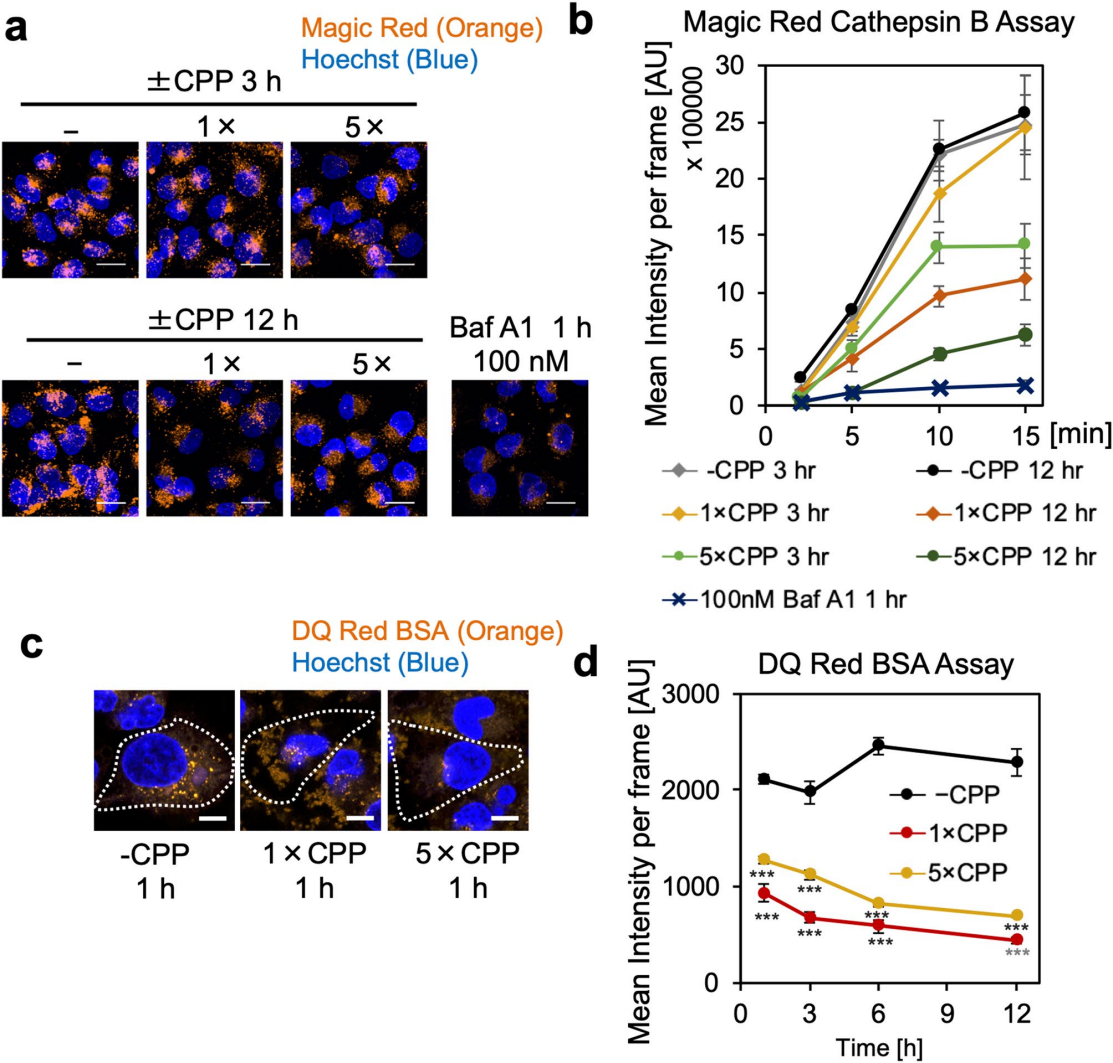
CPPs decrease lysosomal hydrolase activity. **(a)** Microscopic image showing decreased fluorescence intensity of Magic Red, indicating decreased cathepsin B activity in CPP- or BafA1-treated HK-2 cells. We treated HK-2 cells with CPPs or BafA1 for indicated time points, followed by Magic Red cathepsin B assay. Scale bar = 20 μm. **(b)** Line graph showing quantification of the experiment in (**a**). The decrease in cathepsin B activity in CPP-treated HK-2 cells depended on both CPP treatment time and concentration. Data represent the mean intensity of Magic Red calculated from six frames (*n* = 6), expressed as mean ± SEM. **(c)** Microscopic image showing decreased fluorescence intensity of DQ Red BSA, indicating decreased lysosomal hydrolase activity in CPP-treated HK-2 cells. We incubated HK-2 cells with or without CPPs for 1, 3, 6 and 12 h, followed by incubation with DQ Red BSA for 6 h. Scale bar = 10 μm. (**d)** Line graph showing quantification of the experiment in (**c**). Data represent the mean intensity of DQ-Red BSA calculated from six frames (*n* = 6), expressed as mean ± SEM.

As CPP treatment was found to increase luminal pH of LELs by 1.0 unit in HK-2 cells, we examined whether this modest increase affected lysosomal hydrolase activity. First, Magic Red cathepsin B assay was conducted to investigate the effect of increased luminal pH on lysosomal cathepsin B activity in CPP-treated cells. Magic Red penetrates the plasma membrane and produces a strong red fluorescence signal when cleaved by cathepsin B in lysosomes^33^. In CPP-untreated HK-2 cells, we observed a strong fluorescence signal in lysosomes, while CPP treatment decreased the fluorescence (Fig. 7a,b). The decrease in cathepsin B activity in CPP-treated HK-2 cells depended on both the period and concentration of CPP treatment. In addition, the decrease in cathepsin B activity in HK-2 cells treated with CPPs for 3 h was comparable to HK-2 cells treated with 3 nM BafA1 for 1 h (Supplementary Fig. S11), which was similar to the lysosomal alkalization effect of CPPs and BafA1 (Fig. 3f,g).

Hydrolase activity assay using DQ-Red bovine serum albumin (BSA), a membrane-impermeable probe, was also performed to examine the intracellular hydrolase activity in endocytic pathways. Treatment of HK-2 cells with CPPs only for 1 h reduced hydrolase activity in endocytic pathways as estimated by an increase in the fluorescence intensity of hydrolysed DQ Red BSA (Fig. 7c,d). Therefore, a relatively modest increase in pH induced by CPP treatment can decrease hydrolase activity in LELs.

### Lysosomal dysfunction caused by CPPs impairs autophagic flux

**Figure 8:**
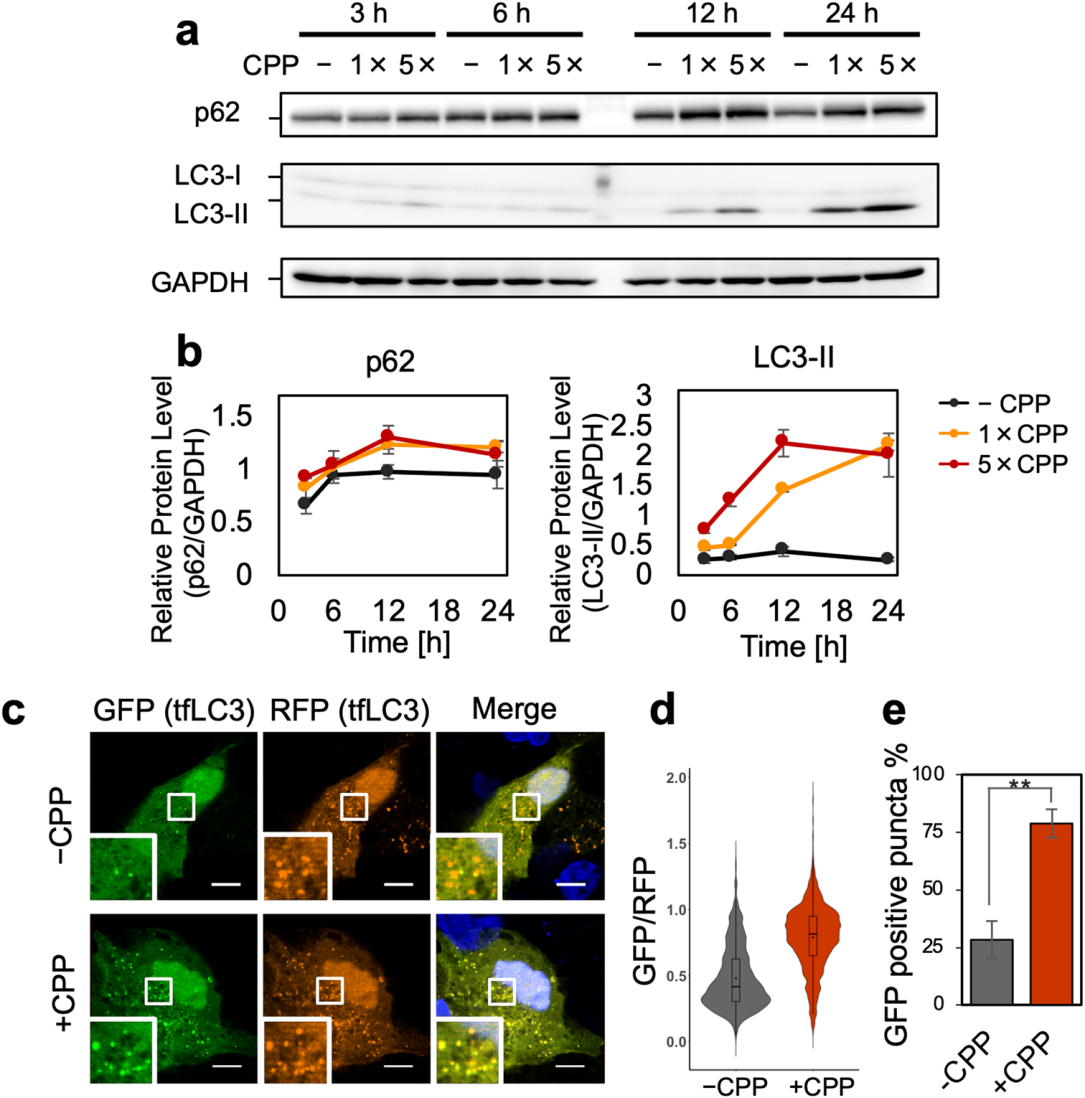
Lysosomal dysfunction due to CPPs induces autophagic flux blockage. **(a)** WB showing p62 and LC3-II accumulated in CPP-treated HK-2 cells. HK-2 cells incubated for 3, 6, 12 and 24 h in low (1×) or high (5×) CPP concentrations was analysed. (**b)** Line graph showing quantification of the bands in (**a**), normalized by GAPDH. Data expressed as mean ± SEM (*n* = 3). Full-length blots are presented in Supplementary Fig. S17. **(c)** Microscopic image showing increased GFP-positive puncta in CPP-treated cell, indicating autophagic flux blockage. We incubated HK-2 cells expressing mRFP-GFP tfLC3 with or without CPPs for 12 h. Scale bar = 10 μm. **(d)** Fluorescence intensities of RFP and GFP quantified for each autophagosome/autolysosome punctum in (**c**). Violin plot showing increased GFP:RFP ratio in CPP-treated HK-2 cells. Data represent results from three frames. We quantified > 800 puncta for each condition. (**e)** Bar graph showing increased GFP-positive puncta in CPP-treated HK-2 cells, indicating autophagic flux blockage. LC3 puncta with GFP:RFP ratio ≧0.6 were considered GFP-positive. Data represent the average of three frames (n = 3), expressed as mean ± SEM. **P < 0.01.

In macroautophagy, autophagosomes are first formed by sequestering membranes, which surround a portion of the cytoplasm and organelles, followed by fusion of lysosomes to degrade the contents^34^. Lysosomal dysfunction due to increased pH inhibits such autophagic flux^35^. Therefore, we examined whether lysosomal dysfunction caused by CPP could also block autophagic flux. WB analysis showed that the amount of p62 and LC3-II gradually increased with incubation time until 12 h and reached a steady state (Fig. 8a–c).

As LC3-II accumulation might be seen not only when autophagy degradation is inhibited but also when autophagy itself is enhanced, mRFP-GFP tandem fluorescent-tagged LC3 (tfLC3) was conducted to determine whether the autophagic flux was blocked^34,36^. In autophagosomes, both GFP and RFP fluorescence is exhibited, whereas in acidified autolysosomes (generated by fusion of autophagosomes with lysosomes), GFP fluorescence is attenuated because of acidic conditions, leaving only the RFP signal to be observed. Figure 8d shows tfLC3-expressing HK-2 cells after 12 h of incubation with or without CPPs. We observed GFP-negative puncta, indicative of acidified autolysosomes seen in normal autophagic flux, mostly in CPP-untreated HK-2 cells and few in CPP-treated HK-2 cells (Fig. 8d). Quantification of each autophagosome/autolysosome punctum showed a higher GFP:RFP ratio in CPP-treated HK-2 cells compared to CPP-untreated HK-2 cells (Fig. 8e). The percentage of GFP-positive puncta was ~ 25% in CPP-untreated HK-2 cells but increased to 75% in CPP-treated HK-2 cells, indicating impaired autophagic flux in CPP-treated cells (Fig. 8e). We also determined whether the autophagic flux impairment was due to defective autophagosome–lysosome fusion or compromised autolysosomal function. Co-localization of LAMP2 and tfLC3 was observed regardless of CPP treatment, indicating that autophagosome–lysosome fusion was not inhibited by CPP (Supplementary Fig. S12). Thus, these results indicated that fusion of autophagosomes with CPP-containing lysosomes failed to acidify the autolysosome, leading to autophagic flux blockage.

### CPPs are weak inducers of non-apoptotic cell death but effective enhancers of H_2_O_2_-induced oxidative stress

**Figure 9:**
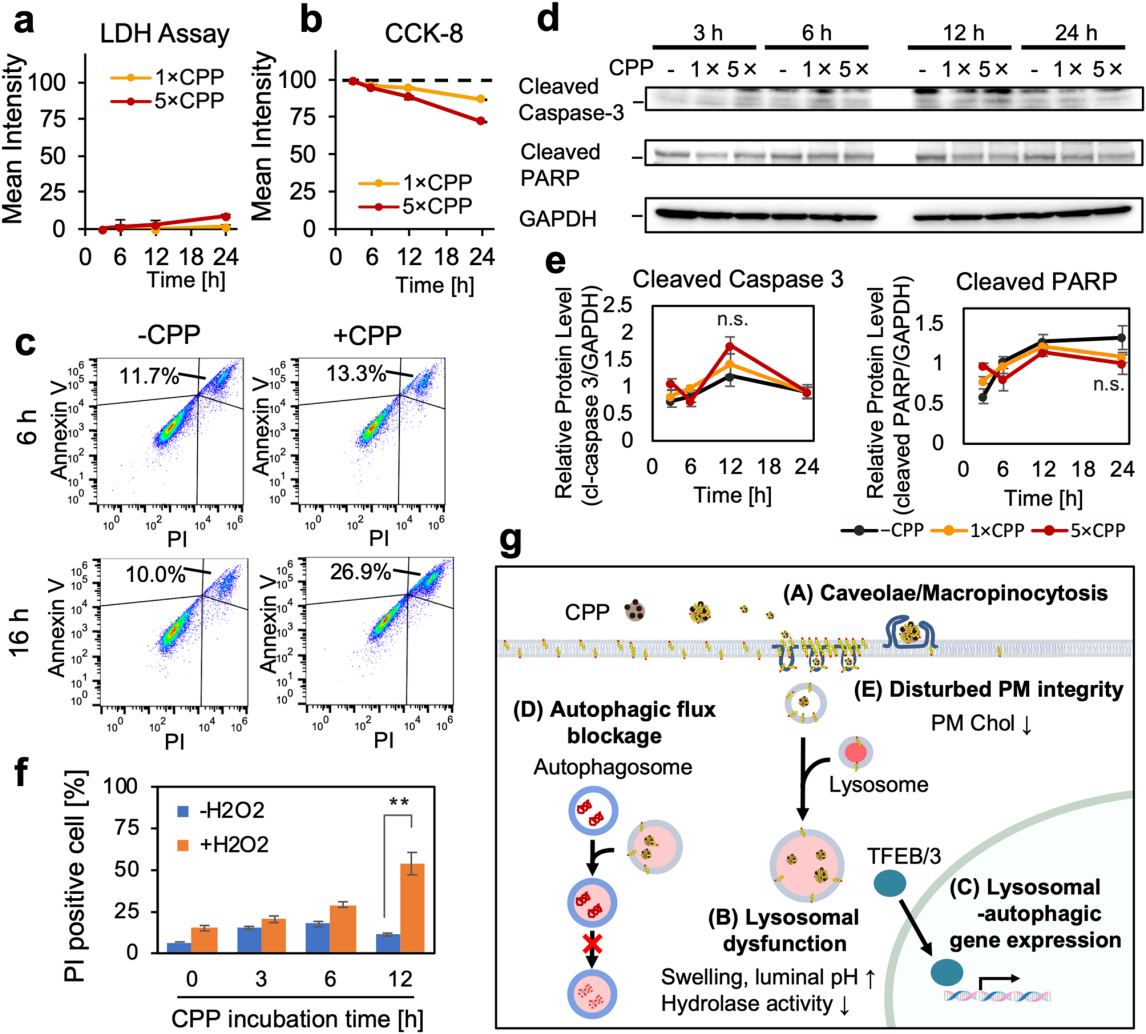
CPPs induce non-apoptotic cell death and make cells susceptible to H_2_O_2_-induced oxidative stress. **(a)** Extracellular LDH quantified for HK-2 cells incubated in low (1×) or high (5×) CPP concentrations for 3, 6, 12 and 24 h. CPP treatment increased LDH release over time. Data expressed as mean ± SEM (*n* = 3). **(b)** Viability quantified for HK-2 cells treated as in (**a**). CPP treatment decreased metabolic activity over time. Data expressed as mean ± SEM (*n* = 3). **(c)** Flow cytometry analysis of Annexin V-PI staining. HK-2 cells incubated with CPP for 6 or 16 h was analysed. Cell death increased (annexin V( +)/PI( +)) over time, but there were no early apoptotic cells (annexin V( +)/PI( −)), indicating non-apoptotic cell death. We used FlowJo software version 10.5.1 (RRID:SCR_008520, Tree Star, https://www.flowjo.com) to analyse data. **(d)** WB showing CPP addition did not increase cleaved caspase-3 or PARP, indicating non-apoptotic cell death. HK-2 cells incubated for 3, 6, 12 and 24 h in low (1×) or high (5×) CPP concentrations were analysed. Full-length blots are presented in Supplementary Fig. S18a. **(e)** Line graph showing quantification of the bands in (**d**), normalized by GAPDH. Data expressed as mean ± SEM (*n* = 3). n.s: not significant. (**f)** Bar graph showing susceptibility of CPP-treated HK-2 cells to H_2_O_2_-induced oxidative stress. We treated HK-2 cells incubated with CPPs for 0, 3, 6 or 12 h with 600 μM H_2_O_2_ and examined cell death by PI staining 12 h later. Data expressed as mean ± SEM (*n* = 3). **P < 0.01. **(g)** Working model of CPP-induced cytotoxicity in HK-2 cells. (A) CPPs internalized via caveola-dependent endocytosis or macropinocytosis. (B) After solubilization under acidic conditions of LELs, CPPs caused LEL swelling and increased luminal pH, decreasing hydrolase activity. (C) Lysosomal dysfunction induced a lysosomal stress response through the TFEB–TFE3 axis, activating lysosomal-autophagic gene transcription. (D) CPPs caused autophagic flux blockage due to lysosomal dysfunction, making cells vulnerable to oxidative stress. (E) CPPs disturbed plasma membrane integrity through reduced plasma membrane Chol. PM, plasma membrane; Chol, cholesterol.

Next, we investigated the effects of lysosomal dysfunction and autophagic flux blockage on cytotoxicity and cell viability using lactate dehydrogenase (LDH) leakage and Cell Counting Kit-8 (CCK-8) cell viability assays. The LDH assay detected a small LDH leakage in the medium, indicating cell death over time, and the effect was relatively pronounced at a high CPP concentration (Fig. 9a). CCK-8 assay showed that the CPP treatment decreased the metabolic activity of cells over time, also significant at a high CPP concentration (Fig. 9b). These results indicated that CPP treatment caused cell death but to a small extent compared to other toxic agents, such as CaPi crystals (Supplementary Fig. S13) or LLME^21,30^.

Next, to elucidate the mode of cell death, we used flow cytometer analysis using annexin V-FITC and propidium iodide (PI) staining^37^. We did not detect annexin V–positive/propidium iodide (PI)-negative cells, indicative of early apoptosis, in HK-2 cells treated with CPPs for 6 or 16 h (Fig. 9c), indicating that CPP-induced cell death is likely to be non-apoptotic. Furthermore, WB analysis using antibodies against cleaved caspase-3 and poly(ADP-ribose) polymerase (PARP), which are apoptosis markers, did not detect activation of the apoptotic pathway. We observed increased caspase-3 and PARP (85 kDa) cleavage (Supplementary Fig. S14a–c) in staurosporine-treated HK-2 cells, a positive control which induces apoptosis, but not in CPP-treated HK-2 cells (Fig. 9d,e). Therefore, we concluded that CPP treatment induces non-apoptotic cell death.

As autophagy is known to protect cells from various insults by digesting damaged proteins and organelles^15^, we next asked if CPP-induced autophagy dysfunction would predispose the cells to cell death caused by oxidative stress. HK-2 cells were incubated with or without CPP for 3–12 h and then exposed to 600 μM H_2_O_2_ to induced oxidative stress. H_2_O_2_-induced cell death in CPP-treated HK-2 cells was significantly enhanced (Fig. 9f), indicating that CPP-treated HK-2 cells are vulnerable to additional oxidative stress, although CPPs alone do not cause effective cell death. With regard to the vulnerability towards oxidative stress, a downregulation was observed of nuclear factor erythroid-2-related factor-2 (Nrf2), which is known to play a critical role in the defence against oxidative stress-induced cytotoxicity^38^ (Supplementary Fig. S15). Although the underlying mechanism of Nrf2 downregulation is unknown, this may be another mechanism that is contributing to the CPP-induced vulnerability towards oxidative stress.

## Discussion

CPPs, a form of nanoparticles comprising CaPi and fetuin-A, are believed to cause renal toxicity^6^. A previous study using artificial CaPi crystals reported the induction of cytotoxic lysosomal membrane permeabilization and ROS generation^8,21^, while serum fetuin-A ameliorated the detrimental effects of CaPi crystals^18,39^. When CaPi crystals are taken up by cells, they dissolve in the acidic environment of lysosomes, increasing the intra-lysosomal osmotic pressure, resulting in lysosomal swelling and lysosomal membrane permeabilization^7^. CPPs cause lysosomal swelling but not lysosomal membrane permeabilization, indicating that the underlying mechanism is similar in part, but the osmotic swelling might be weaker than inorganic CaPi crystals. By inhibiting CaPi crystal growth, fetuin-A might decrease the cytotoxicity of endocytosed CPPs in LELs. Therefore, our findings underscore the importance of the physical properties of CPPs, which are critical for the extent of cytotoxicity.

Although CPPs show only modest cytotoxicity compared to CaPi, CPPs disturb cellular homeostasis in several aspects. In CPP-treated cells, plasma membrane Chol was decreased while lysosomal Chol was increased; this may be because of the binding affinity of CPP for Chol, which could interrupt Chol trafficking and change the Chol distribution between the plasma membrane and lysosomes. The Chol content in the plasma membrane is an important determinant of plasma membrane fluidity and stability that are indispensable for maintaining cellular homeostasis^40,41^. Previous studies have shown that plasma membrane Chol protects proximal tubular cells from ischaemic insults^40^ and that cells treated with MβCD, which depletes Chol from the plasma membrane, show poor plasma membrane repair^22^. These studies along with our own data indicates that CPP-induced reduction in plasma membrane Chol leads to defects in membrane repair and renders the cells vulnerable to mechanical damage.

Interestingly, CPPs disturb lysosomal homeostasis. Following caveola-mediated endocytosis or macropinocytosis, CPPs increase the luminal pH of LELs by ~ 1.0 unit. Unlike many nanoparticles that alkalize LELs and exert strong cytotoxicity via lysosomal membrane permeabilization induction, CPPs induce only a modest increase in the luminal pH, independent of lysosomal membrane permeabilization. Considering the physical properties of CaPi, this luminal pH increase is likely due to solubilization of endocytosed CPPs under endosomal acidic conditions^7,42^. Lysosomal hydrolase activity, which works optimally at low pH, is inhibited following LEL alkalization.

Notably, increased LEL pH due to CPP treatment induces a typical lysosomal stress response through the lysosome-associated mTORC1–TFEB/TFE3 axis. Consistent with our findings, a recent study has shown that lysosomal alkalization using BafA1 and chloroquine inhibits intra-lysosomal proteolysis, decreases mTOR associated with lysosomes, impairs mTORC1 activity and activates TFEB target gene transcription^32^. Sucrose, which accumulates in lysosomes, inactivates mTORC1 without increasing the lysosomal pH, indicating that uncleared material accumulation within lysosomes is the direct cause of mTORC1 inactivation^32^. These findings, along with our own, indicate that lysosomal alkalization by CPP also leads to mTORC1 inactivation, resulting in TFEB/TFE3 target gene up-regulation. Although this up-regulation might relieve CPP-induced lysosomal stress to a degree, it is insufficient to counteract CPP-induced cytotoxicity. Boosting lysosomal exocytosis by TFEB over-expression is effective in lysosomal storage disease (LSD)^43^, so this strategy might also be valid for CPP-induced cytotoxicity.

The detrimental consequence of luminal LEL alkalization is autophagic flux blockage, which we confirmed using mRFP-GFP tandem fluorescent-tagged LC3. CPP-induced LEL alkalization did not inhibit autophagosome–lysosome fusion, and this is consistent with the recent research showing lysosomal acidification is not a prerequisite for fusion^44^. Here, the fusion of autophagosomes with CPP-containing lysosomes suggest that autolysosome acidification is inhibited, leading to autophagic flux blockage.

Autophagy is essential for maintaining mitochondrial quality and proximal tubular function. Interestingly, a study using proximal tubule–specific autophagy-deficient mice showed cellular senescence progression, possibly due to dysfunctional mitochondria accumulation with excessive ROS generation, concomitant with renal dysfunction and fibrosis^14^. Although we expected that CPP-induced inhibition of autophagy flux might also impair the turnover of worn-out mitochondria leading to ROS generation, we did not detect ROS generation under our experimental conditions. It is possible that because CPP-induced inhibition of autophagic flux might be modest, cells might be capable of digesting the damaged mitochondria using the remaining autophagic capacity. Thus, ROS may not be generated, or only an undetectable amount of ROS was generated by incubation with CPP up to 24 h. Prolonged treatment of cells with CPPs is likely required for accumulation of worn-out mitochondria sufficient for detectable ROS generation. However, CPPs induced non-apoptotic cell death to a small extent. Similar to CPPs, fullerenol inhibits autophagic flux and exerts cytotoxicity without inducing oxidative stress in proximal tubular cells^45^. Fullerenol probably first disrupts the actin cytoskeleton, leading to subsequent autophagic flux blockage and mitochondrial dysfunction, resulting in ATP depletion and final cell death^45^. CPPs might exert cytotoxicity through a similar mechanism; however, more extensive studies on the similarity or differences between CPP- and fullerene-mediated cytotoxicity are necessary in order to elucidate the common mechanism underlying NP-mediated cytotoxicity.

Our findings suggest that CPPs exacerbate CKD through decreasing the capacity of autophagy/lysosomal degradation. Proximal tubular cells are susceptible to various insults, including IRI and nephrotoxic agents, such as cisplatin. In these stress conditions, autophagy protect cells by digesting damaged proteins and organelles^15^. A previous study using autophagy-deficient mice showed that IRI induces apoptosis along with accumulation of p62 in proximal tubular cells^16^. In line with this study, although the exact mechanism underlying H_2_O_2_-induced cell death in CPP-treated cells is unclear, CPP-induced inhibition of autophagy flux probably leads to accumulation of worn-out mitochondria in cells, leading to apoptosis. Interestingly, older age and diabetes, both of which is a risk factor of CKD^46^, are associated with autophagy dysfunction. The kidneys of aged mice show a steady increase in autophagy, while further induction of autophagy in response to metabolic stress is blunted^14^. This decreased capacity for additional autophagy stimulation might cause vulnerability to stress conditions, progressing age-related CKD. Another study reported that advanced glycation end products cause diabetic kidney disease by inhibiting the autophagy–lysosomal system^47^. Together with these studies, our findings underscore the importance of the autophagy–lysosomal system as a common factor in CKD, diabetic kidney disease and age-related renal damage.

A limitation of our study is that the precise mechanism leading to cell death remains undetermined. Although we attributed CPP-induced cytotoxicity to alterations in the autophagy–lysosomal system and Chol homeostasis, further research is needed to validate our findings. In addition, other mechanisms that we did not consider in this study might be involved. A recent study has shed light on CPPs as vehicles for various bioactive ligands^48^. In addition to the mineral content of CPP, the effects of other bioactive molecules (e.g. proteins, lipids and nucleic acids) absorbed to the particle surface might induce cytotoxicity. Comparison of the physiochemical properties of endogenous CPP isolated from uraemic serum and CPP synthesized in vitro demonstrated subtle differences in mineral, protein and lipid composition^48^. Thus, characterization of the endogenous CPP in the renal tubules is necessary to better mimic the pathological condition of patients with CKD^48,49^. Although there is limited information regarding the kind and amount of CPP in the proximal renal tubule, we speculate that CPPs bind to the surface of proximal tubule epithelial cells (as seen in Fig. 1) and accumulate with time. Given that with time, CPPs containing amorphous calcium phosphate (primary CPP) mature to CPPs containing more crystalline phases (secondary CPP)^50^, CPPs in the proximal renal tubule could mature into secondary CPPs at the surface of proximal tubule epithelial cell. In addition, compared with CPPs formed in the blood, the presumably low concentration of fetuin-A in the primary urine would likely promote the crystallization of CPP^51^.

Although beyond the scope of the current study, the relationship between CPP and inflammation induction in renal tubules would be of interest to investigate. In macrophages, CPP was shown to induce inflammasome activation and inflammatory cytokine release^52^. As prolonged inflammation promotes fibrosis leading to CKD, CPP-induced inflammation might be another mechanism contributing to CKD progression.

In conclusion, endocytosed CPPs accumulate in LELs and increase their luminal pH by ~ 1.0 unit, resulting in a decrease in lysosomal hydrolase activity and autophagic flux blockage without lysosomal rupture and ROS generation (Fig. 9g). CPP treatment leads to vulnerability to H_2_O_2_-induced oxidative stress and plasma membrane injury, probably because of autophagic flux blockage and decreased plasma membrane Chol, respectively. CPP-induced disruption of lysosomal homeostasis, autophagy flux and plasma membrane integrity might trigger a vicious cycle, leading to progressive nephron loss.

## Methods

### Cell culture and reagents

Human renal proximal tubular epithelial cell line, HK­2 (ATCC CRL­2190) was purchased from American Type Culture Collection (Manassas, USA). We cultured the HK­2 cells in Dulbecco’s Modified Essential Medium and Ham’s F-12 Medium (DMEM/F-12) (#11320082; Gibco, Grand Island, NY, USA) containing 10% foetal bovine serum (FBS) and 100 U/ml of penicillin and 100 μg/ml of streptomycin (#15140-122; Gibco) in 5% CO_2_ at 37 °C. See the Supplementary Information regarding reagents used in this study.

### CPP preparation

Previous studies prepared CPP in various conditions, varying in phosphate and calcium concentration, incubated with serum or with purified fetuin-A alone^52,53^. In this study, we prepared CPPs by mixing 75 μL 1 M CaCl_2_ and 625 μL 200 mM phosphate buffer together in a microcentrifuge tube. We added the resulting CaPi precipitates to 25 mL DMEM/F-12 supplemented with 5% FBS. As the DMEM/F-12 supplemented with 5% FBS itself contains 2 mM calcium and 1 mM phosphate, the final concentration of calcium and phosphate reached 5 and 6 mM, respectively. Next, we incubated the mixture for 24 h at 37 °C and then centrifuged it at 16,000 × *g* for 2 h. After removing the supernatant, we suspended the precipitated CPPs with the same amount (25 mL) of DMEM/F-12 containing 10% FBS to make 1 × CPP, or one-fifth of the initial amount (5 mL), to make 5 × CPP. The total calcium and phosphorus content in 1 × CPP was determined by ICP-MS Nexion 2000 as previously described^54^. 1 × CPP contained 174.8 μg calcium/mL (4.36 mM) with the standard error of mean ± 3.0 and 105.2 μg phosphorus/mL (3.40 mM) with the standard error of mean ± 2.9. The particle size distribution of CPPs was determined by nanoparticle tracking analysis using Nanosight NS300 (Nanosight, Amesbury, UK). CPPs were distributed over 50 ~ 600 nm, and the mode value was 150 nm (see Supplementary Fig. S19). The morphology of CPPs were analysed by transmission electron microscopy. We observed elongate spindle-shaped crystalline CPPs indicative of secondary CPPs (see Supplementary Fig. S19a,b). The fetuin-A presence in CPP was confirmed using SDS-PAGE followed by Coomassie brilliant blue staining (see Supplementary Fig. S19f).

The CaPi crystals were produced with the same protocol with CPP, except that 5% FBS was not added to the DMEM. For fluorescent labelling of CPPs, we added fluorescent bisphosphonate, FITC-alendronate or 5(6)-RhR-dRIS (final concentration 25 nM) to a CaPi mixture and performed the same procedure as before.

### Immunofluorescence confocal microscopy

We seeded HK-2 cells into each well of a 96-well plate. After treatment, we fixed the cells in 4% paraformaldehyde (PFA) for 15 min at room temperature. For LysoTracker staining, we incubated HK-2 cells with 66 nM LysoTracker for 30 min prior to fixation. For immunofluorescence using anti-galectin-3, anti-TFE3 and anti-TFEB antibodies, we permeabilized the fixed cells using 0.2% Triton X-100 in phosphate-buffered saline (PBS) for 3 min at room temperature; permeabilization was not performed in cells stained with LysoTracker or filipin, because permeabilization attenuated the fluorescence. We also confirmed immunofluorescence using anti-LAMP2 and anti-mTOR antibody to be sufficiently stained without permeabilization. For blocking, we incubated HK-2 cells with 3% BSA for 30 min and then incubated them with filipin and/or primary antibodies for > 2 h at 4 °C, followed by incubation with appropriate secondary antibodies for 1 h at room temperature. To stain the nucleus, we incubated the cells with Hoechst 33342 or NucSpot for > 1 h. Finally, we obtained images using a Nikon A1confocal laser scanning microscope (Nikon, Tokyo, Japan). For automated image acquisition using NIS-Elements software Ver4.4 (Nikon, https://www.nikonmetrology.com/en-gb/product/nis-elements-microscope-imaging-software), we used Hoechst 33342 or NucSpot staining for auto-focusing and acquired *z*-stacks. See the Supplementary Information for the details of image analysis.

### Endocytosis assay

We seeded HK-2 cells in 96-well plates and cultured them overnight in a fresh culture medium. Then, we incubated the cells with RhR-CPPs for 12 h, fixed them in 4% PFA and observed them under a Nikon A1confocal microscope. Next, we examined RhR-CPP uptake in the presence of several endocytosis inhibitors in order to identify which endocytic pathway is involved in RhR-CPP uptake. We treated HK-2 cells with 200 μM genistein, 15 μM CPZ or 25 μM EIPA. First, we pre-treated HK-2 cells with the inhibitors for 30 min and then further incubated them with RhR-CPPs in the presence of the inhibitors for 12 h. To distinguish internalized RhR-CPPs from membrane-bound RhR-CPPs, we stained the plasma membrane by immunofluorescence using anti-Cav1 antibody and filipin.

### Transfection

We used FuGENE HD Transfection Reagent for the transfection of Cav1-EGFP and mRFP-GFP tfLC3. HK-2 cells were plated in 8-well chamber slides and cultured them until sub-confluence. Transfection was performed using 400 ng of plasmid DNA, 1.2 μl of transfection reagent and 200 μl of Opti-MEM per well 24 h before experiments.

### Glass bead injury and resealing assay

We incubated HK-2 cells with or without CPPs for 12 h in a 35 mm glass base dish. After washing the cells with PBS, we added 100 μl of growth medium to the dish. Next, we sprinkled acid-washed 425–600 μm glass beads on the cells and gently rocked the dish 10 times to let the beads roll over the cells. Finally, we rinsed the cells in PBS and incubated them in growth medium for 0, 3 and 6 h, observed them under an Eclipse Ts2 microscope and acquired imaged using a DS-Fi3 camera (Nikon).

For the control experiment, we depleted cellular Chol by incubating HK-2 cells with 2% MβCD for 40 min or replenished cellular Chol by further incubation with a medium supplemented with 16 μg/ml of Chol. We performed glass bead injury, as described before, except that we used a medium containing 1 mM calcein to determine the extent of plasma membrane repair following injury.

### Preparation of ratiometric fluorescence pH probes, SiRpH5.5-Dex and SiRpH5-Dex

SiRpH5.5-Dex was prepared by referring to the synthesis of SiRpH5-Dex^28^ as detailed in Supplementary Information.

### pH measurements of LELs using the ratiometric fluorescence pH probes

HK-2 cells were seeded on 8-well chamber slides and cultured for 1 day before imaging. Cells were incubated with or without CPP in DMEM/F12 (10% FBS) containing 0.31 μM (200 μg/mL) ratiometric fluorescence pH probe SiRpH5.5-Dex or SiRpH5-Dex for 6 h at 37 °C, washed three times with DMEM and imaged using a Leica Application Suite Advanced Fluorescence (LAS AF) instrument with a TCS SP5 and 63 × oil immersion objective lens. The light source was a white-light laser SuperK (Leica). The excitation/emission wavelengths were 580 nm and 660 nm/690–750 nm, respectively. After live imaging, the cells were fixed with 4% PFA phosphate buffer solution and an in situ pH calibration was conducted in cells treated with NaPi buffer ranging in pH from 3 to 6.5. pH analysis of the images was performed by Metamorph (Molecular Devices, LLC.), where the pH was quantified from the ratio of the average fluorescence intensity of each ROI, which is the overlap area between the fluorescence images excited at 580 nm and 660 nm.

### Western blotting assay

We lysed HK-2 cells with ice-cold radio-immunoprecipitation assay buffer (1% Triton X-100, 0.1% sodium dodecyl sulphate [SDS], 1% sodium deoxycholate, 150 mM NaCl, 50 mM Tris–HCl [pH 8.0]) with 1 mM PMSF. After centrifugation at 15,000 × *g* for 5 min, we collected the supernatant and measured protein concentrations using the Pierce BCA Protein Assay kit. Next, we boiled the cell lysates in Laemmli’s SDS sample buffer for 5 min, subjected them to 5%–20% gradient sodium dodecyl sulphate polyacrylamide gel electrophoresis (SDS-PAGE) and transferred the SDS-PAGE gels to Immobilon P membranes. Then, we blocked the SDS-PAGE gels in 5% (w/v) powdered milk or BSA, probed them with primary antibodies and incubated them with horseradish peroxidase (HRP)-conjugated secondary antibodies. Finally, we visualized and analysed immune complexes using the Western Lightning Plus-ECL system according to the manufacturer’s instructions (LAS4000 mini; FUJIFILM, Tokyo, Japan).

For lysate dephosphorylation, we added 100 units of calf intestinal alkaline phosphatase (CIAP) to 40 μg of lysate and incubated it for 30 min at 37 °C. The resulting lysate underwent the same procedure as before.

### Intracellular ROS detection

We detected ROS generation using the DCFDA/H2DCFDA kit and analysed it using a fluorescence microscope. Briefly, we incubated HK-2 cells with CPPs for 0, 3, 6 and 12 h and with DCFDA for 30 min. We observed ROS generation by 2 mM LLME treatment for 1 h as a positive control. Finally, we acquired images using a Nikon A1 confocal microscope. For quantification of ROS generation, we analysed the fluorescence intensity of DCFDA probe/cell using NIS-Elements software Ver4.4 (Nikon).

### Lysosomal hydrolase activity assay

We measured cathepsin B activity using the Magic Red cathepsin B detection kit. Briefly, we incubated HK-2 cells with or without CPPs, washed them with PBS and loaded Magic Red cathepsin B reagent for 1–15 min. We used BafA1 treatment as a positive control. Next, we washed the cells with PBS and fixed them in 4% PFA. We acquired images using a Nikon A1 confocal microscope. For quantification of cathepsin B activity, we analysed the fluorescence intensity of Magic Red cathepsin B probe per frame using NIS-Elements software Ver4.4 (Nikon). We used DQ Red BSA to examine intracellular hydrolase activity in endocytic pathways. Briefly, we incubated HK-2 cells with or without CPPs for 1, 3, 6 and 12 h and further incubated them with 10 μg/ml of DQ Red BSA for 6 h in 5% CO_2_ at 37 °C. Next, we washed the cells with PBS and fixed them in 4% PFA. Finally, we acquired images using a Nikon A1 confocal microscope and measured lysosomal hydrolase activity by the fluorescence intensity of DQ Red BSA per frame using NIS-Elements software Ver4.4 (Nikon).

### Autophagic flux assay

We transfected HK-2 cells with mRFP-GFP tfLC3, as described before, followed by incubation with or without CPPs for 12 h. Next, we fixed the cells in 4% PFA, stained the nucleus with Hoechst 33342 and observed the cells under a Nikon A1 confocal microscope. Finally, we quantified the fluorescence intensities of RFP and GFP for each autophagosome/autolysosome punctum and measured the GFP/RFP ratio. LC3 puncta with GFP/RFP ≧0.6 were considered GFP-positive.

### Cell viability and cytotoxicity assay

We incubated HK-2 cells with CPPs for 3, 6, 12 and 24 h in a 96-well plate and quantified cell viability and cytotoxicity using CCK-8(#CK04; Dojindo Molecular Technologies) and LDH assay kit (#CK12; Dojindo Molecular Technologies), respectively, according to the manufacturer’s instructions. See the Supplementary Information for details.

### Apoptosis assay

Apoptosis analysis was performed using the annexin V-FITC kit (#4700; MBL) according to the manufacturer’s instructions. See the Supplementary Information for details.

### H_2_O_2_-induced cell death assay

We incubated HK-2 cells with or without CPPs for 0, 3, 6 and 12 h and subsequently exposed them to 600 μM H_2_O_2_ to induce oxidative stress. We examined cell death by PI staining 12 h later. We stained the cells with 3.3 μg/ml of PI and 0.1 mg/ml of Hoechst 33342 for 1 h at 37 °C to label dead and all cells, respectively. We acquired images using a Nikon A1 confocal microscope and calculated the percentage of PI-positive cells using NIS-Elements software Ver4.4 (Nikon).

### Statistical analysis

We assessed the statistical significance of differences from the control using Dunnett’s test as a multiple comparison test, and the data between the two groups were evaluated by Welch’s t-test. Results are reported as mean ± standard error of the mean (SEM). *P* < 0.05 was considered statistically significant. The number of samples used is shown in each figure legend.

## Supplementary information

Supplementary Information.
