# Supplementary material for: Calciprotein particle-induced cytotoxicity via lysosomal dysfunction and altered cholesterol distribution in renal epithelial HK-2 cells
Source: Sci Rep. 2020 Nov 18;10:20125. doi: 10.1038/s41598-020-77308-3 (PMC7676272; doi:10.1038/s41598-020-77308-3)
Supplement: Supplementary file 1 — Supplementary Information. [file 41598_2020_77308_MOESM1_ESM.docx]

**Supplementary Information**

**Calciprotein Particle–Induced Cytotoxicity via Lysosomal Dysfunction and Altered Cholesterol Distribution in Renal Epithelial HK-2 Cells**

*Rina Kunishige^1^, Mai Mizoguchi^2^, Asako Tsubouchi^1^, Kenjiro Hanaoka^2^, Yutaka Miura^3^, Hiroshi Kurosu^3^, Yasuteru Urano^2, 4, 5^, Makoto Kuro-o^3^, and Masayuki Murata^1, 6*^*

^1^ Department of Life Sciences, Graduate School of Arts and Sciences, The University of Tokyo, 3-8-1 Komaba, Meguro-ku, Tokyo, 153-8902, Japan

^2^ Graduate School of Pharmaceutical Sciences, The University of Tokyo, 7-3-1 Hongo, Bunkyo-ku, Tokyo 113-0033, Japan

^3^ Division of Anti-aging Medicine, Center for Molecular Medicine, Jichi Medical University, 3311-1 Yakushiji, Shimotsuke, Tochigi 329-0498, Japan.

^4^ Graduate School of Medicine, The University of Tokyo, 7-3-1 Hongo, Bunkyo-ku, Tokyo 113-0033, Japan

^5^ CREST (Japan) Agency for Medical Research and Development (AMED), 1-7-1 Otemachi, Chiyoda-ku, Tokyo 100-0004, Japan

^6^ Cell Biology Center, Institute of Innovative Research, Tokyo Institute of Technology, 4259 Nagatsuta, Midori-ku, Yokohama, Kanagawa, 226-8503, Japan

* Correspondence should be addressed to Masayuki Murata (email: [mmurata@bio.c.u-tokyo.ac.jp](mailto:mmurata@bio.c.u-tokyo.ac.jp) )

INDEX

[**1.** **Supplementary Figures** 2](#_Toc43807859)

[**2.** **Supplemental Methods** 31](#_Toc43807860)

[**3.** **Supplemental Scheme** 39](#_Toc43807861)

[**4.** **Supplemental References** 47](#_Toc43807862)

[**5.** **Appendix** Photophysical properties of the pH probes 48](#_Toc43807863)

1. **Supplementary Figures**


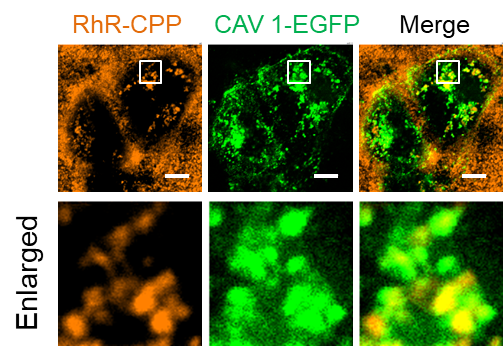


**Supplementary Fig. S1: RhR-CPPs are co-localized with caveolin-1.**

Microscopic image showing co-localization of RhR-CPPs with Cav1-EGFP.

We incubated HK-2 cells transfected with Cav1-EGFP with RhR-CPPs for 12 h. Scale bar = 10 μm. RhR-CPPs, 5(6)-RhR-dRIS-labelled calciprotein particles; Cav1, caveolin-1; EGFP, enhanced green fluorescent protein.

1×　 5×

1×　 5×

in −FBS

in +FBS

Cholesterol bound to CPP


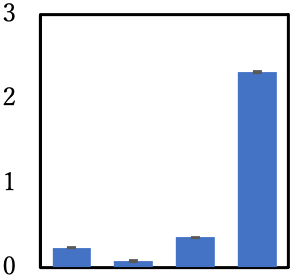


Relative Cholesterol Level

*

*

*

**Supplementary Fig. S2: CPPs have binding affinity towards Chol.**

Quantitation of the amount of Chol bound to CPPs using the Cholesterol Quantitation Kit. We incubated CaPi (crystals/amorphous) in a medium with or without 10% FBS for 24 h, centrifuged it at 16,000 ×*g* and measured the Chol bound to the precipitates. Serum Chol bound to CPPs and co-precipitated upon centrifugation. Data represent mean ± SEM (*n* = 3). ****P* < 0.001. CPPs, calciprotein particles; Chol, cholesterol; CaPi, calcium phosphate; SEM, standard error of the mean.

**
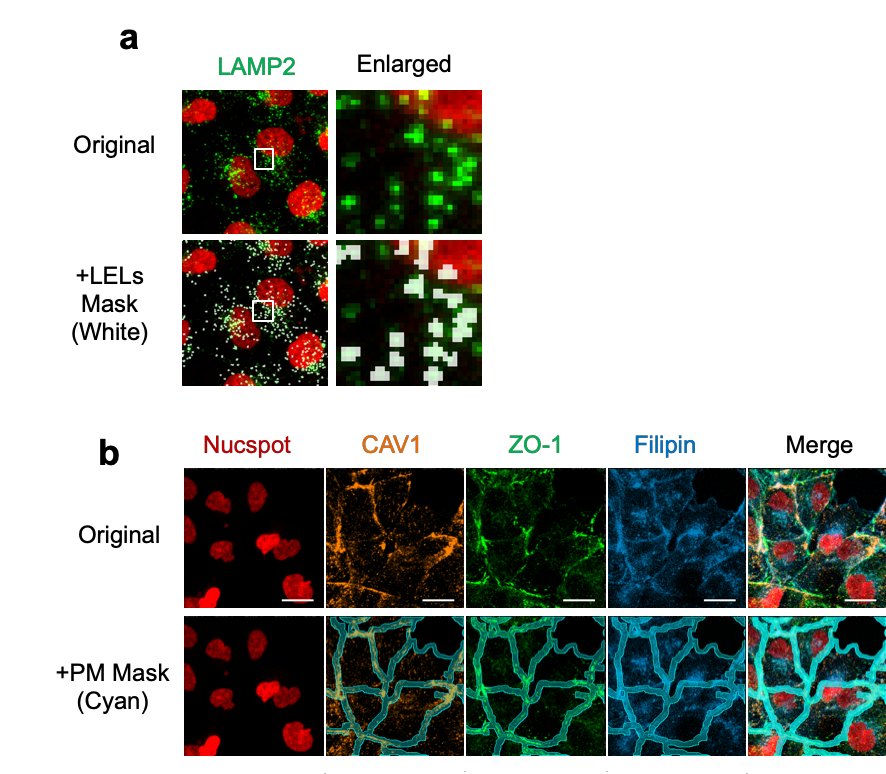
**

**Supplementary Fig. S3: Computer algorithm–defined LEL and plasma membrane regions.**

**a,** LAMP2 channel used to define the LEL region. (Upper panel) Original image. (Lower panel) White masks represent the computer-identified LEL region. **b,** Cav1 and ZO-1 channels used to define the plasma membrane region. (Upper panel) Original image. (Lower panel) Cyan masks represent the computer-identified plasma membrane region. We used NIS-Elements software Ver4.4 (Nikon, RRID:SCR_014329, https://www.nikonmetrology.com/en-gb/product/nis-elements-microscope-imaging-software) to analyse data. LELs, late endosomes/lysosomes; PM, plasma membrane; LAMP2, lysosomal-associated membrane protein 2; Cav1, caveolin-1.


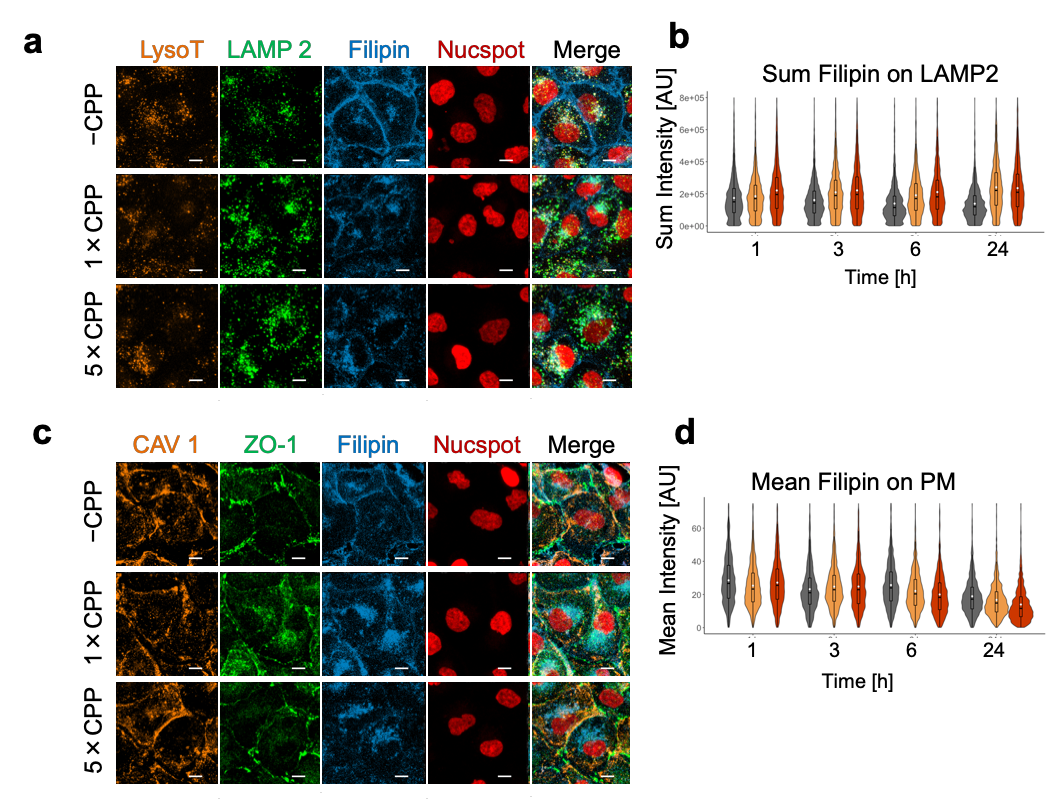


**Supplementary Fig. S4: Plasma membrane** **Chol decreases concomitantly with an increase in LELs.**

**a,** Confocal microscopy images showing Chol accumulation in LELs in CPP-treated HK-2 cells. We fixed HK-2 cells incubated for 1, 3, 6 and 24 h in low (1x) or high (5x) CPP concentrations in 4% PFA. We performed Chol staining using filipin in parallel with immunofluorescence using LAMP2 antibody in order to define the LEL region. in CPP-treated HK-2 cells. Scale bar = 10 μm. **b,** Violin plot showing increased Chol in LELs in CPP-treated HK-2 cells. We calculated the sum intensity of filipin fluorescence on the LAMP2-positive region/cell. **c,** Confocal microscopy images showing Chol decrease in CPP-treated HK-2 cells. We fixed HK-2 cells incubated for 1, 3, 6 and 24 h in low (1x) or high (5x) CPP concentrations in 4% PFA. We performed Chol staining using filipin in parallel with immunofluorescence using Cav1 and ZO-1 antibody in order to define the plasma membrane region. Scale bar = 10 μm. **d,** Violin plot showing decreased plasma membrane Chol in CPP-treated HK-2 cells. We calculated the mean intensity of filipin fluorescence on the plasma membrane region/cell. PM, plasma membrane; Chol, cholesterol; LELs, late endosomes/lysosomes; PFA, paraformaldehyde; LAMP2, lysosomal-associated membrane protein 2; Cav1, caveolin-1.


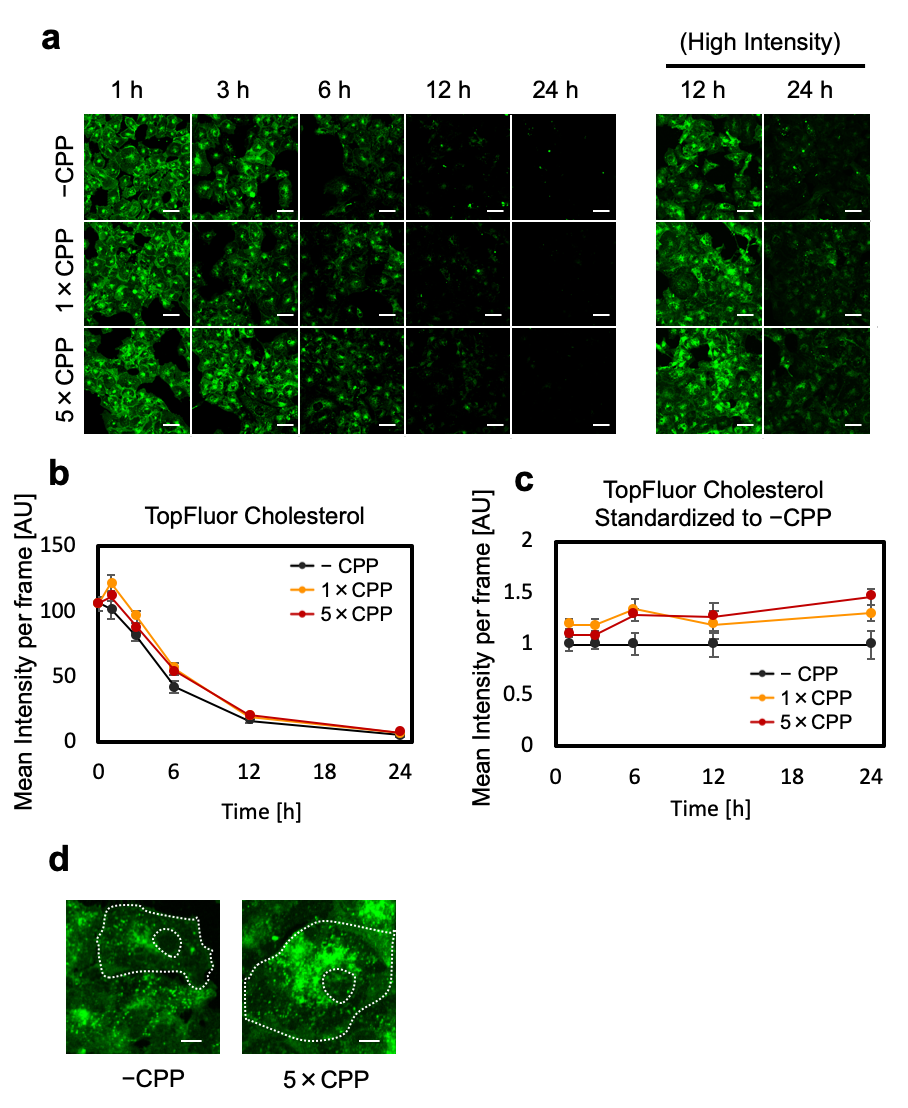


**Supplementary Fig. S5: CPPs slightly delay TF-Chol efflux.**

**a,** Confocal microscopy images showing gradual efflux of TF-Chol. We pulse-labelled plasma membrane Chol with TF-Chol and incubated HK-2 cells with or without CPPs for 1, 3, 6, 12 and 24 h. TF-Chol was internalized and then gradually exported from the cells; almost all of the TF-Chol was exported in ~24 h. Scale bar = 50 μm. **b,** Line graph showing a slight delay in extra-cellular efflux of TF-Chol in the presence of CPPs. Data represent results from eight images (*n* = 8), expressed as mean ± SEM. **c,** The same data in (**b**) standardized to the −CPP condition for each time point, showing TF-Chol accumulation in the presence of CPPs. Data represent results from eight images (*n* = 8), expressed as mean ± SEM. **d,** Enlarged image of HK-2 cells showing increased TF-Chol accumulation in peri-nuclear vesicles. We incubated HK-2 cells for 24 h with or without CPPs. White dotted lines outline the cell and the nucleus. Scale bar = 10 μm. CPPs, calciprotein particles; PM, plasma membrane; Chol, cholesterol; TF-Chol, TopFluor cholesterol; SEM, standard error of the mean.


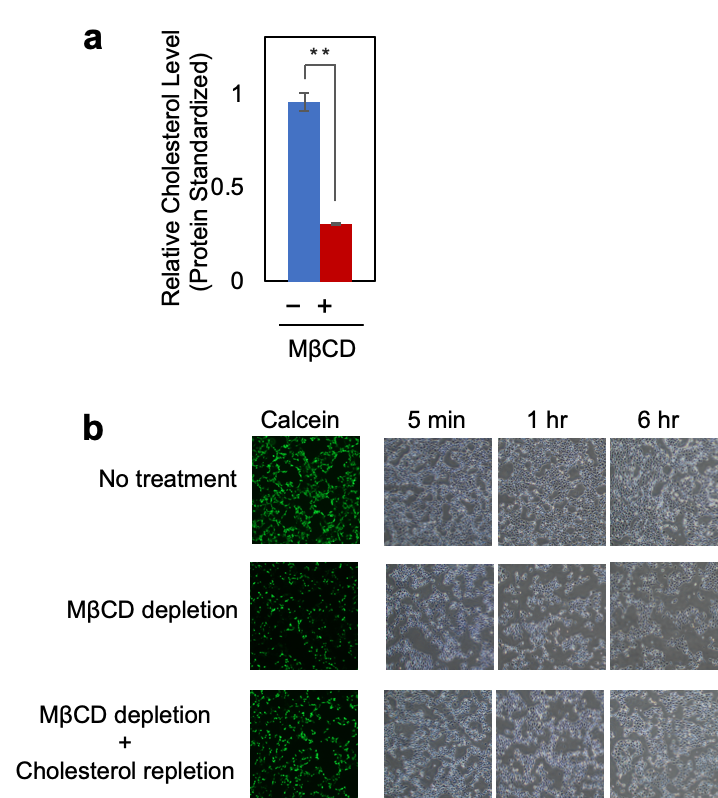


**Supplementary Fig. S6: Plasma membrane** **Chol depletion by MβCD makes cells susceptible to plasma membrane injury.**

**a,** Chol quantification of HK-2 cells which underwent plasma membrane Chol depletion by incubation with 2% MβCD for 20 min. We quantified total Chol and normalized the values to total protein content. MβCD treatment substantially depleted cellular Chol. Data represent mean ± SEM (*n* = 3). ***P* < 0.01.

**b,** Microscopic image showing that Chol depletion increased cell vulnerability to plasma membrane damage. We subjected HK-2 cells with depleted or repleted Chol to plasma membrane damage using 425–600 μm glass beads. We performed glass bead injury in a medium containing calcein in order to determine the extent of plasma membrane repair following injury. Calcein entered cells through plasma membrane breaks and was retained in the cytosol following membrane resealing. In HK-2 cells with depleted Chol, plasma membrane repair was dampened, calcein continually leaked out and was not retained in the cells, leading to significant cell death. Cells which underwent Chol repletion after MβCD depletion showed improved plasma membrane repair and cell layer recovery compared to Chol-depleted HK-2 cells. PM, plasma membrane; Chol, cholesterol; MβCD, methyl-β-cyclodextrin; SEM, standard error of the mean.


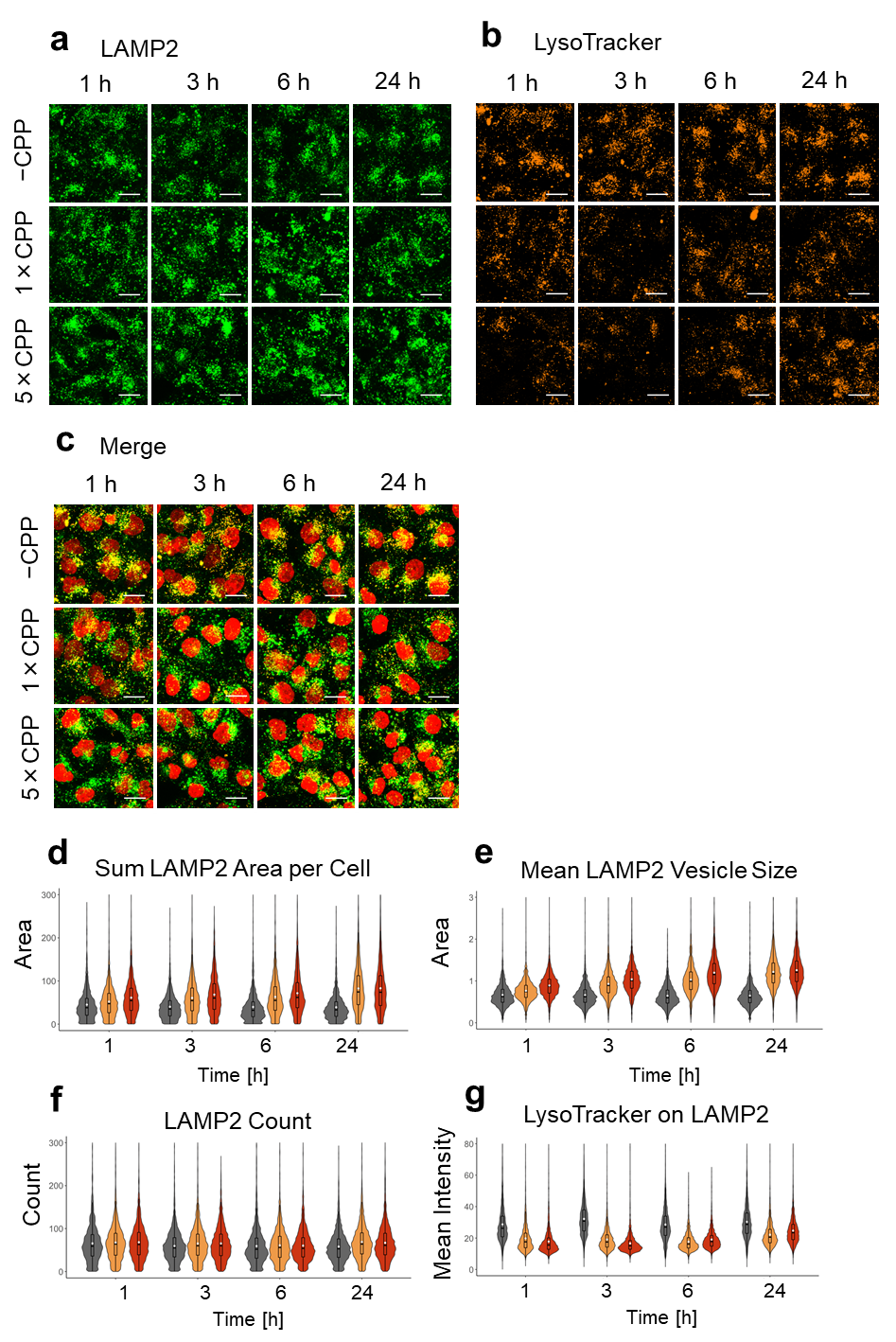


**Supplementary Fig. S7: CPPs induce lysosomal swelling and luminal pH increase of LELs.**

**a,** Confocal microscopy images showing LAMP2 staining in cells treated with or without CPPs. We stained HK-2 cells incubated for 1, 3, 6 and 24 h in low (1×) or high (5×) CPP concentrations with LysoTracker and then fixed them in 4% PFA. We performed immunofluorescence using LAMP2 antibody in order to define LEL region. Scale bar = 20 μm. **b,** Representative image of LysoTracker. CPP addition decreased fluorescence, indicating a pH increase. Scale bar = 20 μm. **c,** Merge image of LAMP2 (green), LysoTracker (orange) and nuclear-staining NucSpot (red). Scale bar = 20 μm. **d,** Violin plot showing increased LAMP2 area in CPP-treated HK-2 cells. The sum LAMP2 area was calculated per cell. **e,** Violin plot showing increased LAMP2 vesicle size in CPP-treated HK-2 cells. The mean LAMP2 vesicle size was calculated per cell. **f,** Violin plot showing a slight increase in the LAMP2 vesicle number 24 h after CPP addition. The mean number of LAMP2 vesicles was calculated per cell. **g,** Violin plot showing decreased fluorescence intensity of LysoTracker on the LAMP2 region in CPP-treated HK-2 cells. We calculated the mean fluorescence intensity of LysoTracker on the LAMP2 region/cell. CPPs, calciprotein particles; LELs, late endosomes/lysosomes; PFA, paraformaldehyde; LAMP2, lysosomal-associated membrane protein 2.

**
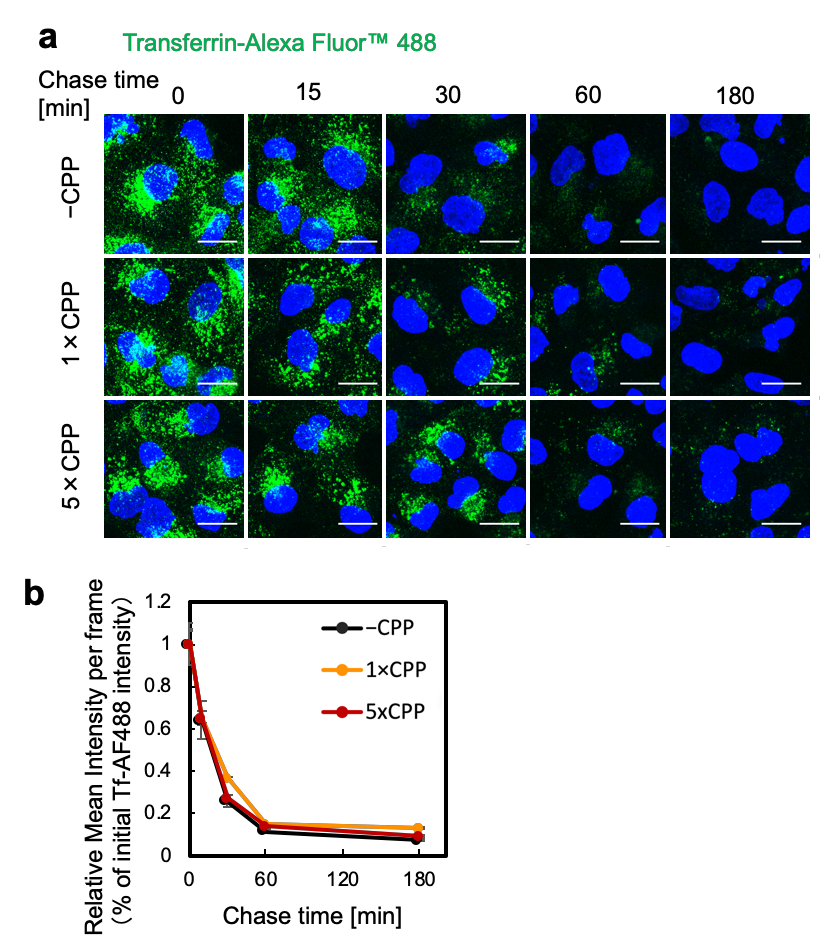
**

**Supplementary Fig. S8: CPPs do not affect transferrin recycling.**

**a,** Pulse-chase study using Tf-AF488 showing unaltered recycling in CPP-treated HK-2 cells. We incubated HK-2 cells with or without CPPs for 24 h, followed by pulse-labelling with 10 μg/ml of Tf-AF488 for 30 min. Then, we chased the cells with 10 μg/ml of unlabelled transferrin for 0, 15, 30, 60 and 180 min and fixed them in 4% PFA. Tf-AF488 was gradually exocytosed from the cells regardless of CPP addition. Scale bar = 20 μm. **b,** Line graph showing the relative mean fluorescence intensity of Tf-AF488 in the cells in (**a**). The Tf-AF488 exocytosis rate was unaltered in HK-2 cells with or without CPPs. Data represent results from six images (*n* = 6), expressed as mean ± SEM. CPPs, calciprotein particles; Tf-AF488, transferrin–Alexa Fluor 488; PFA, paraformaldehyde; SEM, standard error of the mean.


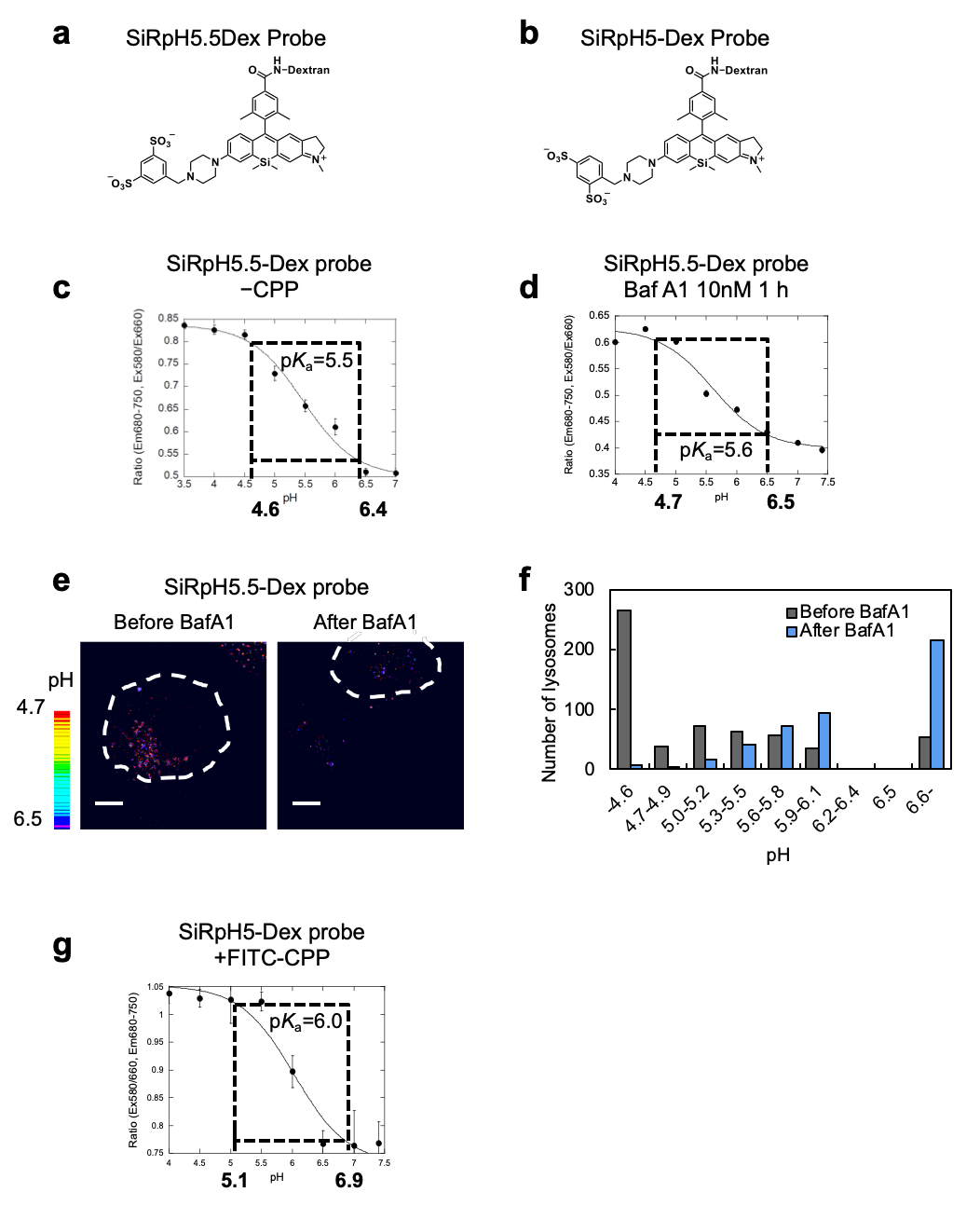


**Supplementary Fig. S9: Measurement of absolute pH using new ratiometric probes, SiRpH5.5-Dex and SiRpH5-Dex.**

**a,** Structure of the SiRpH5.5-Dex probe. **b,** Structure of the SiRpH5-Dex probe. **c,** Calibration curve in HK-2 cells incubated with SiRpH5.5-Dex in the absence of CPPs for 6 h. We fixed the cells and performed in situ pH calibration by treating them with NaPi buffer (pH range: 3.0 to 6.5). The p*K*_a_ was 5.5, and pH measurement between 4.6 and 6.4 was confirmed to be valid. **d,** Calibration curve of SiRpH5.5-Dex in HK-2 cells incubated with SiRpH5.5-Dex in the absence of BafA1. This calibration curve was used for the experiment with the cells incubated with BafA1 for 1 h. The p*K*_a_ was 5.6, and pH measurement between 4.7 and 6.5 was confirmed to be valid. **e,** Microscopic ratio image of the SiRpH5.5-Dex probe before (left) and after (right) BafA1 addition. White dotted lines outline the cell. Scale bar = 10 μm. **f,** Histogram showing the quantified pH of >450 LELs under each condition in (**e**). We calculated the pH of individual LELs using the calibration curve in (**d**). BafA1 treatment increased the mean pH from 5.14 to 6.11. **g,** Calibration curve in HK-2 cells incubated with SiRpH5-Dex for 6 h. This calibration curve was used for the pH determination in Fig. 4c. The p*K*_a_ was 6.0, and pH measurement between 5.1 and 6.9 was confirmed to be valid.


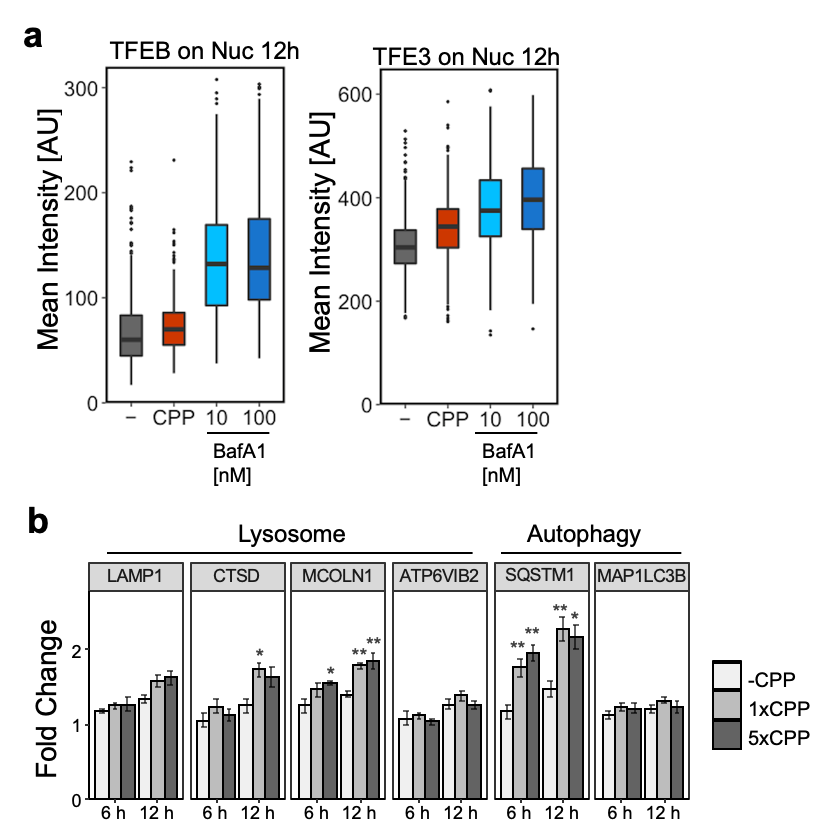


**Supplementary Fig. S10: CPPs trigger TFEB-/TFE3-mediated lysosomal stress response.**

**a,** Boxplot showing increased nuclear intensity of TFEB/TFE3 in CPP- and BafA1-treated HK-2 cells. We treated HK-2 cells with CPPs or BafA1 for 12 h, followed by immunofluorescence using TFEB/TFE3 antibody. The box plot represents the mean fluorescence intensity of TFEB/TFE3 in the nucleus. **b,** Bar graph showing increased expression of autophagy and lysosome–related genes in CPP-treated cells. We treated HK-2 cells with low (1×) or high (5×) CPP concentrations for 6 or 12 h and analysed changes in the expression of TFEB/TFE3 target genes by qPCR. CPPs significantly increased the expression of *LAMP1*, *CTSD*, *MCOLN1* and *SQSTM1* (*n* = 4). **P* < 0.05, ***P* < 0.01. CPPs, calciprotein particles; TFEB, transcription factor EB; TFE3, transcription factor E3; BafA1, bafilomycin A1; qPCR, quantitative PCR.


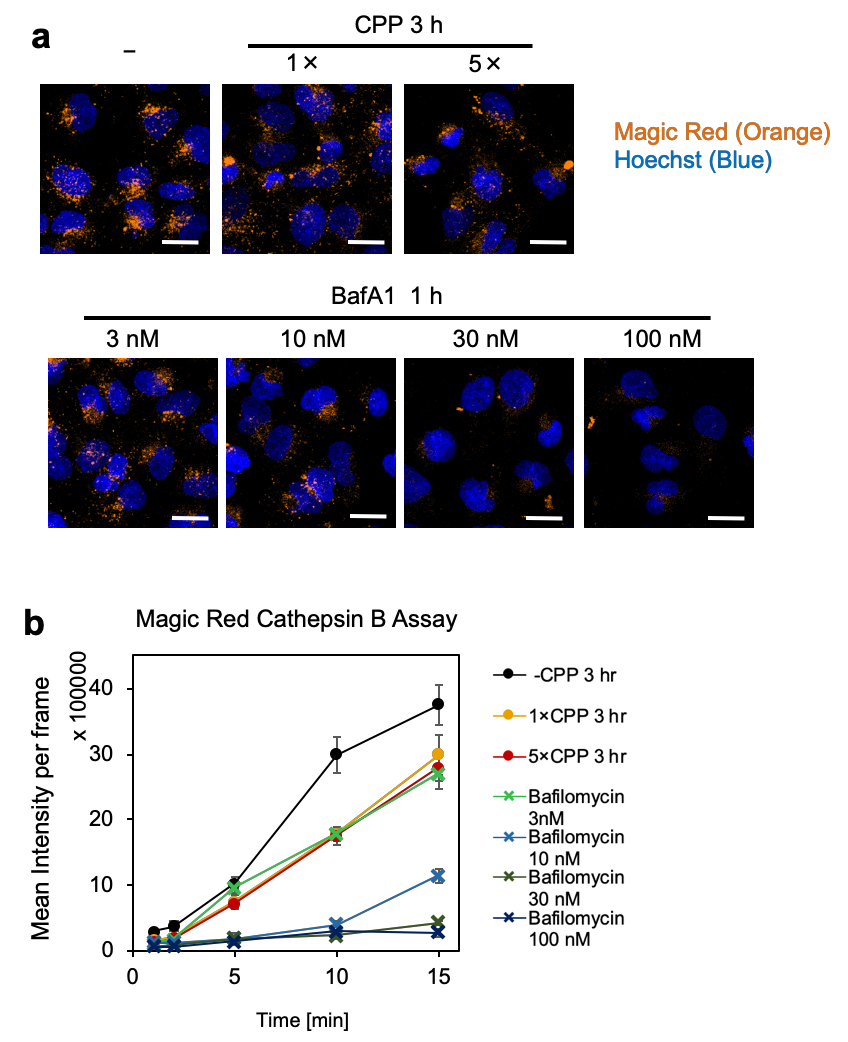


**Supplementary Fig. S11: CPP treatment for 3 h decreases cathepsin B activity comparable to 3 nM BafA1 treatment for 1 h.**

**a,** Confocal microscopy images showing decreased fluorescence intensity of Magic Red, indicating decreased cathepsin B activity in CPP- or BafA1-treated HK-2 cells.

We performed Magic Red cathepsin B assay using samples treated with CPPs for 3 h or BafA1 for 1 h. Scale bar = 20 μm. **b,** Line graph showing quantification of the experiment in (**a**). Data represent the mean fluorescence intensity of Magic Red calculated from six frames (*n* = 6), expressed as mean ± SEM. The decrease in the fluorescence intensity of Magic Red in CPP-treated HK-2 cells was comparable to cells treated with 3 nM BafA1. CPPs, calciprotein particles; BafA1, bafilomycin A1; SEM, standard error of the mean.


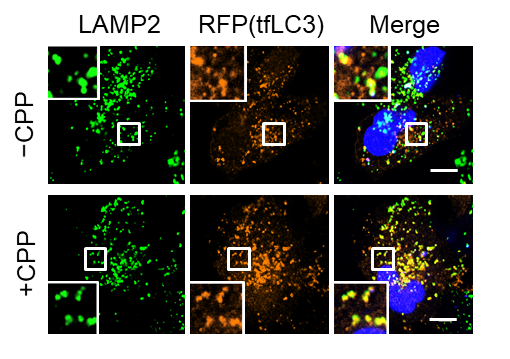


**Supplementary Fig. S12: CPP does not inhibit autophagosome–lysosome fusion.**

Microscopic image showing co-localization of LAMP2 and mRFP-GFP tfLC3, indicating autophagosome–lysosome fusion. We incubated HK-2 cells expressing tfLC3 with or without CPPs for 12 h followed by fixation and immunofluorescence using anti-LAMP2 antibody. Co-localization of LAMP2 and mRFP-GFP tfLC3 was observed regardless of CPP treatment. Scale bar = 20 μm.


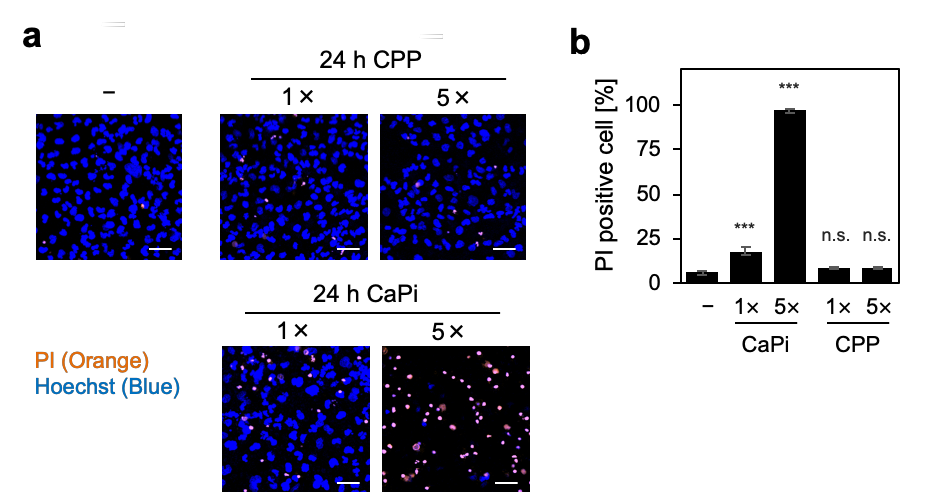


**Supplementary Fig. S13: CaPi crystals induce greater degree of cell death compared to CPP**

**a,** Microscopic image showing increased PI staining, indicating cell death in cells treated with CaPi crystals. We treated HK-2 cells with CPPs or CaPi for 24 h, followed by PI and Hoechst staining. Scale bar = 50 μm **b,** Bar graph showing quantification of the experiment in (**a**). Data represent results from three images (*n* = 3), expressed as mean ± SEM. ****P* < 0.001, n.s.: not significant. SEM, standard error of the mean.


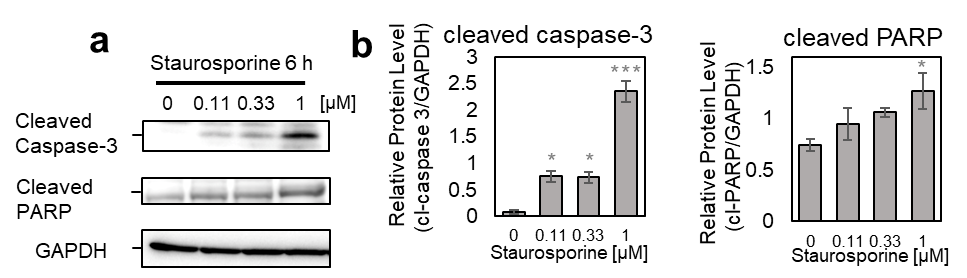


**Supplementary Fig. S14: Caspase-3 and PARP are cleaved upon apoptosis induction.**

**a,** WB showing increased cleaved caspase-3 and PARP in HK-2 cells treated with staurosporine, a positive control to induce apoptosis. **b,** Bar graph showing quantification of the bands in (**a**), normalized by GAPDH. Full-length blots are presented in Supplementary Fig. S18b. Data are expressed as mean ± SEM (*n* = 3). **P* < 0.05, ****P* < 0.001. PARP, poly(ADP-ribose) polymerase; GAPDH, glyceraldehyde 3-phosphate dehydrogenase; SEM, standard error of the mean.


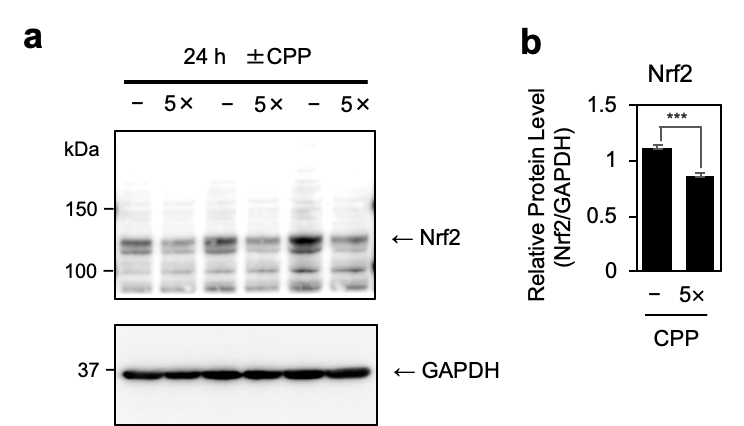


**Supplementary Fig. S15: CPP treatment for 24 h decreases Nrf2 protein level.**

**a,** WB showing decreased Nrf2 in HK-2 cells treated with CPP for 24 h. **b,** Bar graph showing quantification of the bands in (**a**), normalized by GAPDH. Data are expressed as mean ± SEM (*n* = 3). ****P* < 0.001. GAPDH, glyceraldehyde 3-phosphate dehydrogenase; SEM, standard error of the mean.


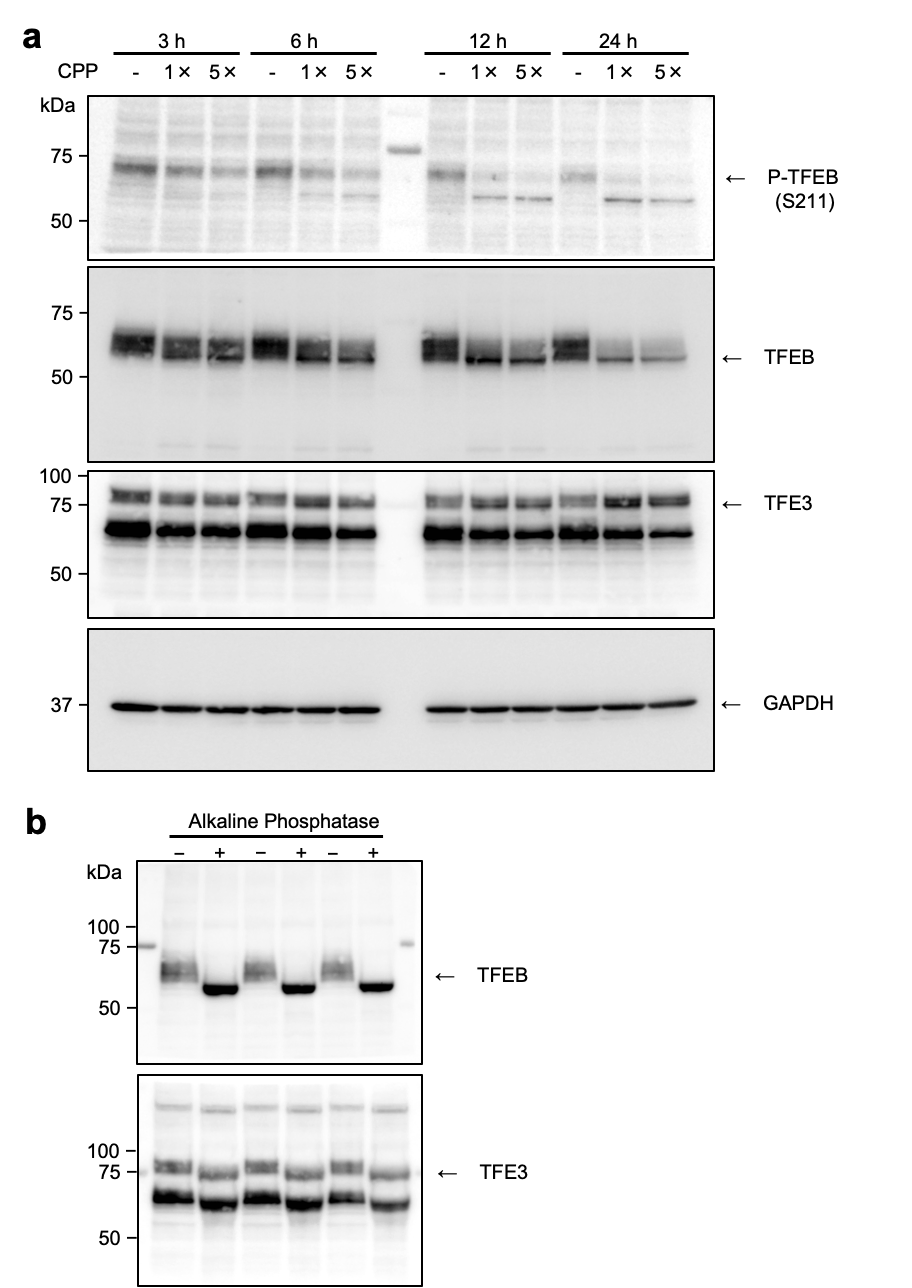


**Supplementary Fig. S16: Full-length Western blot images**

**a,** Full-length blot image for Fig. 6c. **b,** Full-length blot image for Fig. 6d.

**
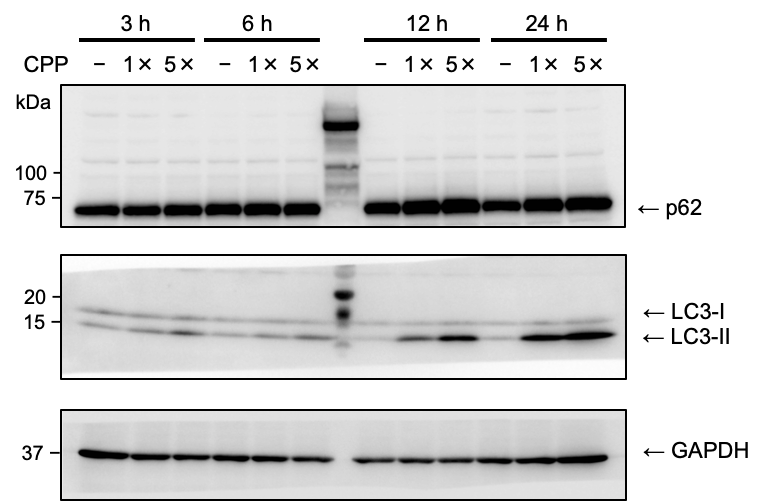
**

**Supplementary Fig. S17: Full-length Western blot image for Fig. 8a.**

**
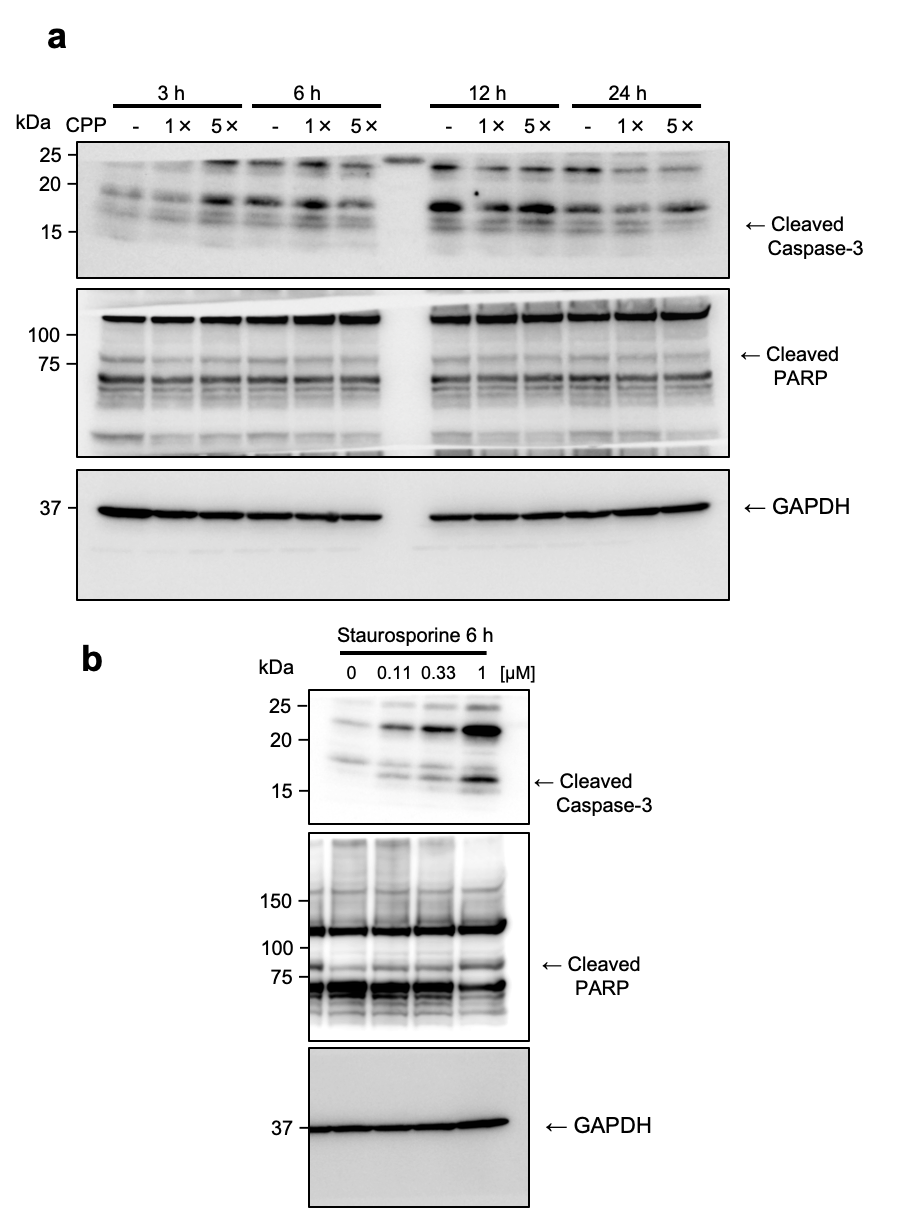
**

**Supplementary Fig. S18: Full-length Western blot images**

**a,** Full-length blot image for Fig. 9d. **b,** Full-length blot image for Supplementary Fig. S13a.

**
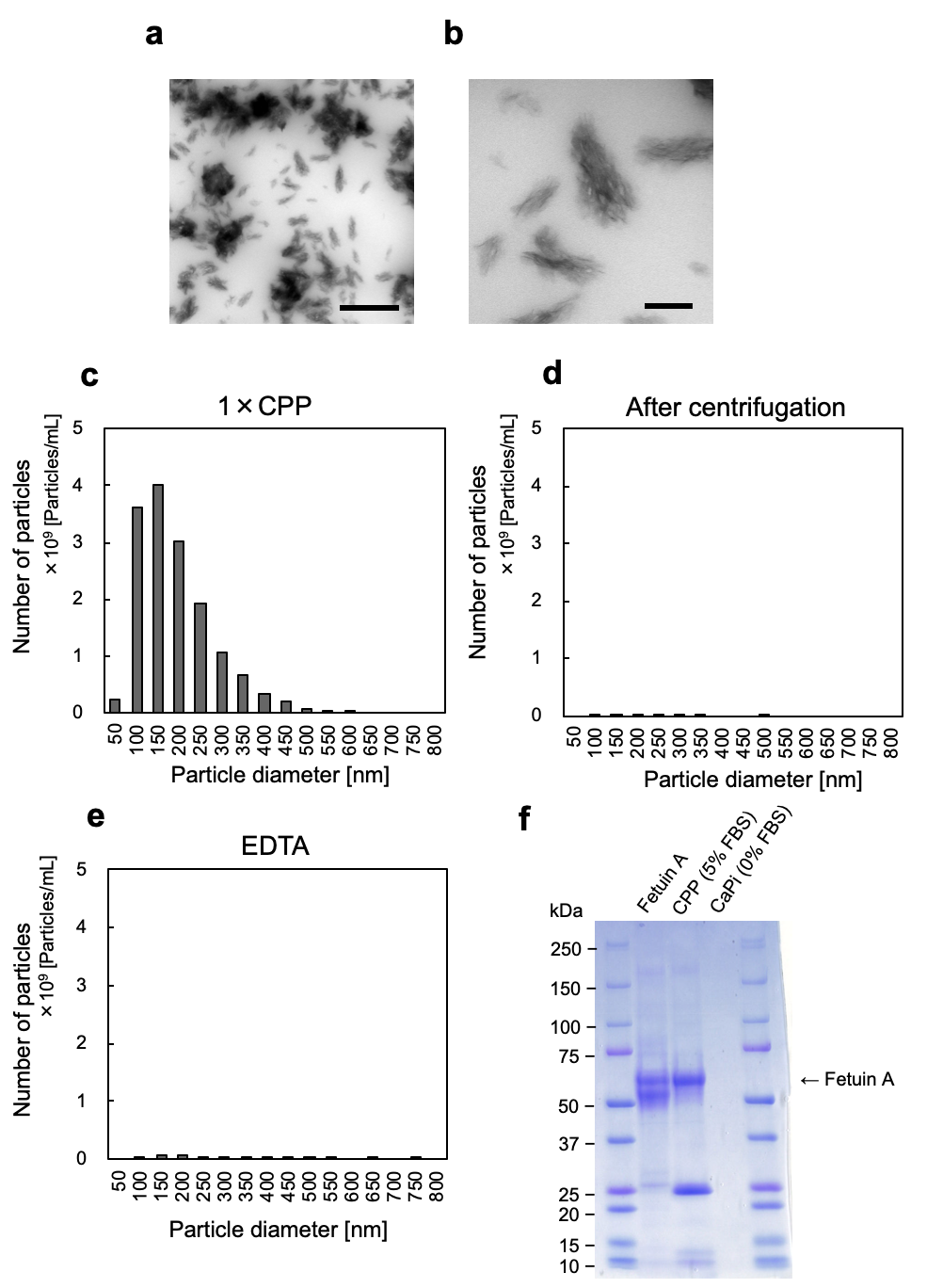
**

**Supplementary Fig. S19: Characterization of the CPP used in this study**

**a,** Transmission electron microscopic observation of the CPP used in this study. Bar = 500 nm. **b,** Enlarged image of CPP in (**a**). Elongate spindle-shaped crystalline CPPs were observed, indicative of secondary CPPs. Bar = 100 nm. **c,** The particle size distribution of 1×CPP used in this study. **d,** 1× CPP was centrifugated at 24,000×*g* for 30 minutes and the supernatant was subjected to nanoparticle tracking analysis. **e,** 1× CPP was treated with EDTA (final concentration at 50 mM) and subjected to nanoparticle tracking analysis. The number of particles dramatically decreased, indicating CPP dissolution by EDTA. **f,** Presence of fetuin-A in CPP was confirmed by Coomassie brilliant blue staining. CPP contained a protein band around 60 kDa, which was identical with the band size of fetuin-A.

**Supplementary Table 1: Primer oligonucleotides used for real-time PCR**

| **Gene** | **Forward primer** | **Reverse primer** |
| --- | --- | --- |
| LAMP1 | ACGTTACAGCGTCCAGCTCAT | TCTTTGGAGCTCGCATTGG |
| CTSD | AACTGCTGGACATCGCTTGCT | CATTCTTCACGTAGGTGCTGGA |
| MCOLN1 | TTGCTCTCTGCCAGCGGTACTA | GCAGTCAGTAACCACCATCGGA |
| ATP6V1B2 | GAGGGGCAGATCTATGTGGA | GGCTTCTTCTCCAACGACAG |
| SQSTM1 | CTGCCTCCTGGTCTCTTCAC | GTTAGGAGGGACAGGGTTCC |
| MAP1LC3B | ACCATGCCGTCGGAGAAG | ATCGTTCTATTATCACCGGGATTTT |
| GAPDH | GCCATCAATGACCCCTTCATTGACC | CGCCTGCTTCACCACCTTCTTGATG |

1. **Supplemental Methods**

**2.1. Reagents**

Filipin (#F9765), chlorpromazine (#C8138), EIPA(5-(N-Ethyl-N-isopropyl) amiloride) (#A3085), genistein (#G6649), methyl-β-cyclodextrin (MβCD) (#C4555), phenylmethylsulphonyl fluoride (PMSF), cholesterol (#C4951), glass beads (#G8772, acid-washed, 425-600 μm), Cholesterol Quantitation Kit (#MAK043), fetuin from fetal bovine serum (#F3004) were purchased from Sigma-Aldrich (St Louis, MO, USA). 96-well plates were purchased from Corning (#4517; NY, USA) or greiner Bio-One (#655090; Tokyo, Japan). Glass base dishes (35mm) were purchased from IWAKI (#3911-035; Chiba, Japan). Six-well dishes (#140675), 60mm dishes (#150462) and Lab-Tek 8-well chamber slides (#155411) were purchased from NUNC. Immobilon-P PVDF Membrane (#IPVH00010) and 0.22 μm Millex-GV Filter (#SLGV004SL) were purchased from Millipore (MA, USA). Lysotracker Red DND-99 (#L7528) and Opti-MEM (#31985070) were purchased from Invitrogen (CA, USA). L-leucyl-L-leucine, methyl ester, monohydrochloride (LLME) (#16008) and staurosporine (#81590) were purchased from Cayman Chemical (Ann Arbor, MI, USA). Hydrogen Peroxide (H2O2) (#081-04215) and 5–20% precast polyacrylamide gels SuperSep Ace (#197-15011, 197-15021) were purchased from Wako (Osaka, Japan). Magic Red Cathepsin B Assay (#937) was purchased from Immunochemistry Technologies (MN, USA). DQ Red BSA (#D12051) and Pierce BCA Protein Assay kit (#23225) was purchased from ThermoFisher (MA, USA). NucSpot Live Cell Nuclear Stain (#40082) was purchased from Biotium (CA, USA). Calcein (#340-00433), Hoechst 33342 (#H342), and Cell Counting Kit-8(CCK-8)/ Cytotoxicity LDH Assay Kit-WST (#CK04) were purchased from Dojindo (Kumamoto, Japan). TopFluor Cholesterol (#810255P) was purchased from Avanti Polar Lipids (AL, USA). 5(6)-RhR-dRIS (#BV140301) was purchased from BioVinc (CA, USA). Propidium iodide (PI) (#P3566) was purchased from Molecular Probes (OR, USA). Bafilomycin A1(Baf-A1) (#S1413) was purchased from Selleck (TX, USA). Alkaline phosphatase (CIAP) (#2250A) was purchased from Takara (Tokyo, Japan). MEBCYTO Apoptosis Kit (Annexin V-FITC Kit) (#4700) was purchased from MBL (Nagoya, Japan). FuGENE HD Transfection Reagent (#E2311) was purchased from Promega (WI, USA). RNeasy Mini Kit (#74106) was purchased from Qiagen (GmBH, Germany). ReverTra Ace qPCR RT Kit (#FSQ-101) was purchased from Toyobo (Kagoshima, Japan). Fast SYBR Green Master Mix (#4385612) was purchased from Applied Biosystems (CA, USA). DCFDA / H2DCFDA - Cellular Reactive Oxygen Species Detection Assay Kit (#ab113851) was purchased from Abcam (MA, USA). Western Lightning Plus-ECL was purchased from PerkinElmer (MA, USA).FITC-alendronate was synthesized by amine coupling between FITC-carboxylic acid and the primary amine in alendronate^1^. ptfLC3 (pmRFP-EGFP-rLC3) plasmid was a gift from Dr. Tamotsu Yoshimori, in Osaka University^2,3^. We used the following primary antibodies: anti–human galectin-3 (#556904; BD Pharmingen, CA, USA); anti-phospho-TFEB (Ser211) (#37681; CST, MA, USA); anti-TFE3 (#HPA023881; Sigma-Aldrich, MO, USA); anti-TFEB (CST, #4240); anti-Caveolin-1 (CST, #3267); anti-PARP (#9532); anti-PARP (CST, #9532); anti-cleaved Caspase-3 (CST, #9661); anti-mTOR (CST, #2983); anti-LC3 (MBL, #M152-3 for IF, #PM036 for WB, Nagoya, Japan); anti-p62 (MBL, #PM045); anti-Nrf2 (MBL, #PM069); anti-LAMP2 (#h4b4: Hybridoma Bank, IA, USA); GAPDH (Millipore, #MAB374); anti-cleaved Caspase-3 (CST, #9661); mouse anti‐ZO‐1 antibody was a gift from Dr. Mikio Furuse in National Institute for Physiological Sciences, Aichi, Japan^4^. We used the following secondary antibodies: HRP-linked anti-rabbit IgG (CST, #7074); HRP-linked anti-mouse IgG (Promega, #W402B); Alexa Fluor 488 anti-mouse (Invitrogen, #A-11001); Alexa Fluor 546 anti-mouse (Invitrogen, #A-11030); Alexa Fluor 647 anti-mouse (Invitrogen, #A-21236); Alexa Fluor 488 anti-rabbit (Invitrogen, #A-11034); Alexa Fluor 546 anti-rabbit (Invitrogen, #A-11035); Alexa Fluor 647 anti-rabbit secondary antibody (Invitrogen, #A-21245).

**2.2. Image analysis**

We analysed all confocal images using NIS-Elements software Ver4.4 (Nikon, RRID:SCR_014329). For z-stack images, we performed maximum intensity projection prior to subsequent image analysis. Next, we detected areas of the nucleus using binary images in the Hoechst or NucSpot channel, areas of the cell membrane using Cav1 and ZO-1 channels and LEL areas using the LAMP2 channel. We measured the mean or sum fluorescence intensity on the region of interest and analysed the size and number of LAMP2 vesicles using the LAMP2 channel. For co-localization analysis of mTOR and LAMP-2, we calculated Pearson’s correlation coefficient using the NIS-Elements software Ver4.4 (Nikon, RRID:SCR_014329).

**2.3. Cholesterol quantitation**

We measured the amount of Chol bound to CPPs, or the total Chol content of HK-2 cells, using the Cholesterol Quantitation Kit (MAK043; Sigma-Aldrich). Briefly, we incubated CaPi precipitates in a medium with or without 10% serum for 24 h, then centrifuged them at 13,000 ×*g* for 2 h and washed the precipitates with a medium without serum. After centrifugation at 13,000 ×*g* for 30 min, we discarded the supernatant, extracted the sample using chloroform:isopropanol:NP-40 (7:11:0.1) in a micro-centrifuge tube and spun it at 13,000 ×*g* for 10 min to remove insoluble material. Next, we air-dried the organic phase at 50°C to remove chloroform, followed by speed-vacuuming for 30 min to remove any residue organic solvent. Finally, we dissolved the dried lipids using 200 μl of cholesterol assay buffer for Chol quantification and measured the fluorescence intensity at an excitation wavelength of 530 nm and an emission wavelength of 590 nm.

**2.4. Cholesterol metabolism assay**

To investigate Chol trafficking from LELs to other organelles or the plasma membrane, we mixed 50 μg/ml of TF-Chol and 10 mg/ml of MβCD in a serum-free medium, sonicated the mixture at 37°C to solubilize Chol and then filtered it through a 0.22 μm polyvinylidene difluoride (PVDF) filter. Next, we added the solution to HK-2 cells and incubated them for 20 min at 37°C to pulse-label the plasma membrane. Subsequently, we washed the cells thrice with PBS, replaced the culture medium with fresh medium and further incubated the cells with or without CPPs for 1, 3, 6, 12 and 24 h. Finally, we acquired images using a Nikon A1confocal microscope and analysed the mean fluorescence intensity of TF-Chol per frame using NIS-Elements software Ver4.4 (Nikon, RRID:SCR_014329, RRID:SCR_014329).

**2.5. Transferrin recycling assay**

We incubated HK-2 cells with or without CPPs for 24 h, followed by pulse-labelling with 10 μg/ml of transferrin–Alexa Fluor 488 (Tf-AF488) for 30 min. Next, we chased the cells with 10 μg/ml of unlabelled transferrin for 0, 15, 30, 60 and 180 min and fixed them in 4% PFA. We acquired images using a Nikon A1confocal microscope and measured the fluorescence intensity of Tf-AF488 using NIS-Elements software Ver4.4 (Nikon).

**2.6. mRNA extraction and RT-PCR**

We purified total RNA from HK-2 cells using the RNeasy Mini Kit (Qiagen, Hilden, Germany) and reverse-transcribed this with the ReverTra Ace quantitative reverse transcription polymerase chain reaction (qRT-PCR) kit (Toyobo, Osaka, Japan). For real-time PCR, we used the fast SYBR Green Master Mix (Applied Biosystems, Foster City, CA, USA) and the StepOnePlus Real-Time PCR System (Applied Biosystems). Supplementary Table 1 lists the primer oligonucleotides used for real-time PCR. We used glyceraldehyde 3-phosphate dehydrogenase (GAPDH) as an internal standard.

**2.7. Cell viability and cytotoxicity assay**

We incubated HK-2 cells with CPPs for 3, 6, 12 and 24 h in a 96-well plate and quantified cell viability using CCK-8 assay (CCK-8, #CK04; Dojindo Molecular Technologies) according to the manufacturer’s instructions. Briefly, we incubated CPP-treated HK-2 cells with 10 μl of CCK-8 solution and 100 μl of fresh medium for 1 h at 37°C. We measured absorbance at a wavelength of 450 nm using an Epoch 2 micro-plate spectrophotometer (BioTek, Winooski, VT, USA). For background subtraction of CPP turbidity, we also measured absorbance at a wavelength of 630 nm and subtracted it from absorbance at a wavelength of 450 nm. Viability in CPP-untreated HK-2 cells was considered 100%.

We quantified cytotoxicity using the LDH assay kit (Cytotoxicity LDH Assay Kit-WST, #CK12; Dojindo Molecular Technologies) according to the manufacturer’s instructions. Briefly, we transferred the supernatant from the wells of CPP-treated HK-2 cells to a new 96-well clear plate. We added 100 μl of Working Solution and incubated the plate for 30 min at 37°C. Next, we added 50 μl of Stop Solution and measured absorbance at a wavelength of 490 nm using an Epoch 2 micro-plate spectrophotometer (BioTek). LDH release in CPP-untreated HK-2 cells was considered 0%, while that in lysis buffer–treated cells was considered 100%.

**2.8. Apoptosis assay**

Apoptosis analysis was performed using the annexin V–FITC kit (#4700; MBL) according to the manufacturer’s instruction. Briefly, we incubated 2.0 × 105 HK-2 cells with or without CPPs for 6 or 16 h and then re-suspended them in 85 μl of binding buffer. Next, we added 10 μl of annexin V–FITC and 5 μl of PI (100 μg/ml), incubated the cells for 15 min in the dark at room temperature, added 400 μl of binding buffer and analysed the cells by flow cytometry (SH800; Sony Corporation, Japan). For each condition, we acquired 10,000 gated cells. We used FlowJo software version 10.5.1 (RRID:SCR_008520, Tree Star, https://www.flowjo.com) to analyse data. Cells stained positive for annexin V–FITC and negative for PI were considered early apoptotic, while cells stained positive for both PI and annexin V–FITC were considered non-viable, either apoptotic or non-apoptotic.

**2.9. Coomassie brilliant blue staining of protein bound to CPP**

CPP and CaPi was prepared as described in CPP preparation in the main text. For positive control, CPP was prepared by incubating with purified fetuin from fetal bovine serum (final concentration of 0.5 mg/mL) instead of 5% FBS. 1 mL 1×CPP or 1×CaPi was centrifuged at 16,000 ×g for 2 h. After removing the supernatant, we suspended the precipitated CPPs with ice-cold radio-immunoprecipitation assay buffer (1% Triton X-100, 0.1% sodium dodecyl sulphate [SDS], 1% sodium deoxycholate, 150 mM NaCl, 50 mM Tris-HCl [pH 8.0]) with 1 mM PMSF. After centrifugation at 15,000 ×g for 5 min, we collected the supernatant and boiled the lysates in Laemmli’s SDS sample buffer for 5 min, subjected them to 5%–20% gradient sodium dodecyl sulphate polyacrylamide gel electrophoresis (SDS-PAGE). Gels were stained with Coomassie brilliant blue for 1 hour and destained in 25% ethanol/7.5% acetic acid for 1 hour, followed by several changes of water.

**2.10. Electron Microscopy**

The CPP samples were absorbed to formvar film coated nickel grids and were dried at room temperature. The CPPs were visualized without staining using the transmission electron microscope (JEM-1400Plus; JEOL Ltd., Tokyo, Japan) at an acceleration voltage of 80 kV. Digital images (3296 × 2472 pixels) were taken with a CCD camera (EM-14830RUBY2; JEOL Ltd.).

**Supplemental Scheme**

Synthetic scheme for **SiRpH5.5**.

***tert*-Butyl 4-(3-bromophenyl)piperazine-1-carboxylate (2)**

To a solution of 1-bromo-3-iodobenzene (**1**) (450 µL, 3.5 mmol) in toluene (15 mL) were added *N*-Boc-piperazine (657 mg, 3.5 mmol), Pd(OAc)_2_ (79 mg, 0.35 mmol), BINAP (220 mg, 0.35 mmol) and Cs_2_CO_3_ (5.77 g, 17.6 mmol) under argon atmosphere. The reaction mixture was stirred for 18 h at 85℃. The reaction mixture was diluted with NaHCO_3_ aq., and extracted with CH_2_Cl_2_. The combined organic layers were washed with brine, dried over Na_2_SO_4_ and concentrated to dryness. The residue was purified by column chromatography (NH silica gel, AcOEt/*n*-hexane) to afford *tert*-butyl 4-(3-bromophenyl)piperazine-1-carboxylate (**2**) (641.3 mg, 1.89 mmol, 53%).

^1^H-NMR (300 MHz, CDCl_3_) δ: 1.48 (s, 9H), 3.12-3.14 (t, *J*=4.9, 4H), 3.55-3.57 (t, *J*=5.1, 4H), 6.81-6.84 (dd, *J*=8.3, 1H), 6.97-6.99 (dd, *J*=4.4, 1H), 7.02-7.04 (t, *J*=2.0, 1H), 7.09-7.13 (t, *J*=8.0, 1H); ^13^C-NMR (75 MHz, CDCl_3_) δ: 28.3, 48.8, 79.9, 114.8, 119.2, 122.7, 123.2, 130.3, 152.4, 154.6.

**6-Bromo-1-methylindoline (5)**

6-Bromoindoline (**4**) (1.57 g, 80 mmol), paraformaldehyde (2.40 g, 80 mmol), and NaBH_3_CN (1.26 g, 40 mmol) in AcOH (40 mL) was stirred for 3 h at r.t., and the reaction was quenched by adding NaOH aq. The whole was extracted with CH_2_Cl_2_, and washed with brine. The organic layer was dried over Na_2_SO_4_ and evaporated to dryness. Purification of the residue by column chromatography (silica gel, AcOEt/*n*-hexane) provided pure 6-bromo-1-methylindoline (**5**) (1.03 g, 4.85 mmol, 60%).

^1^H-NMR (300 MHz, CDCl_3_) δ: 2.71 (s, 3H), 2.84-2.89 (t, *J*=8.3, 2H), 3.28-3.34 (t, *J*=9.2, 2H), 6.53 (s, 1H), 6.72-6.75 (dd, *J*=2.7, 1H), 6.86-6.89 (d, *J*=2.9, 1H); ^13^C-NMR (75 MHz, CDCl_3_) δ: 28.1, 35.5, 56.0, 109.9, 119.9, 120.9, 125.1, 129.2, 154.7; LRMS (ESI^+^): [M+H]^+^, 212.

***tert*-Butyl 4-{3-[dimethyl(1-methylindolin-6-yl)silyl]phenyl}piperazine-1-carboxylate (7)**

To a 100 mL well-dried flask flushed with argon, 6-bromo-1-methylindoline (**5**) (695 mg, 3.3 mmol) and THF (30 mL) were added. After the solution was cooled to -78℃, *sec*-BuLi (3.3 mL, 1 M, 3.3 mmol) was added. After the reaction mixture was stirred at the same temperature for 15 min, the resulting solution was transferred via cannula to a solution of dichlorodimethylsilane (4.5 mL, 16.5 mmol) in THF (5 mL). And this solution was stirred for 1 h at -78℃. The solvent and the redundant dichlorodimethylsilane were removed by evaporation and the resulting **6** was dried *in vacuo* for 1 h. To a 100 mL well-dried flask flushed with argon, **2** (1.02 g, 3.0 mmol) and THF (30 mL) were added. After the solution was cooled to -78℃, *sec*-BuLi (3 mL, 1 M, 3.0 mmol) was added. After the reaction mixture was stirred at the same temperature for 15 min, the resulting mixture was transferred via cannula into the solution of **6** in THF (30 mL) at -78℃. The reaction mixture was warmed to room temperature and stirred for 1 h. Then, the reaction was quenched with water (10 mL) and extracted with CH_2_Cl_2_. The organic layer was dried over Na_2_SO_4_ and evaporated to dryness. Purification of the residue by column chromatography (silica gel, AcOEt/*n*-hexane) provided pure *tert*-butyl 4-{3-[dimethyl(1-methylindolin-6-yl)silyl]phenyl}piperazine-1-carboxylate (**7**) (904 mg, 2.01 mmol, 67%).

^1^H-NMR (300 MHz, CDCl_3_) δ: 0.51 (s, 6H), 1.48 (s, 9H), 2.73 (s, 3H), 2.91-2.95 (t, *J*=11.7, 4H), 3.09-3.11 (t, *J*=4.9, 1H), 3.25-3.29 (t, *J*=8.3, 1H), 3.55-3.57 (t, *J*=4.9, 1H), 6.61 (s, 1H), 6.83-6.84 (d, *J*=2.9, 4H), 6.90-6.93 (dd, *J*=8.8, 2.9, 1H), 7.06-7.08 (t, *J*=2.9, 1H), 7.11 (d, *J*=1.5, 4H), 7.24-7.27 (d, *J*=8.8, 1H); ^13^C-NMR (75 MHz, CDCl_3_) δ: -2.0, 28.4, 28.7, 36.1, 49.6, 55.9, 76.6, 79.8, 112.1, 117.3, 122.5, 123.9, 124.1, 126.3, 128.5, 131.7, 136.7, 139.7, 150.6, 152.8, 154.7.

***tert*-Butyl 4-{4-bromo-3-[(5-bromo-1-methylindolin-6-yl)dimethylsilyl]phenyl}piperazine-1-carboxylate (8)**

To a solution of *tert*-Butyl 4-{3-[dimethyl(1-methylindolin-6-yl)silyl]phenyl}piperazine-1-carboxylate (**7**) (162 mg, 0.36 mmol) in 3 mL CH_3_CN was added NH_4_OAc (5.5 mg, 0.070 mmol) at 0℃. The reaction mixture was stirred for 10 min, then, *N*-bromosuccinimide (NBS) (133.2 mg, 0.75 mmol) was added to the solution. The mixture was stirred for 30 min at 0℃. The reaction mixture was warmed to room temperature and stirred for 2 h. The reaction mixture was diluted with NaHCO_3_ aq., and extracted with CH_2_Cl_2_. The combined organic layers were washed with brine, dried over Na_2_SO_4_ and evaporated to dryness. The residue was purified by column chromatography (silica gel, AcOEt/*n*-hexane) to afford *tert*-Butyl 4-{4-bromo-3-[(5-bromo-1-methylindolin-6-yl)dimethylsilyl]phenyl}piperazine-1-carboxylate (**8**) (204 mg, 0.33 mmol, 93％).

^1^H-NMR (300 MHz, CDCl_3_) δ: 0.72 (s, 6H), 1.47 (s, 9H), 2.70 (s, 3H), 2.91-2.95 (t, J=11.7, 2H), 3.03-3.05 (t, J=4.9, 4H), 3.28-3.32 (t, J=8.3, 3H), 3.53-3.56 (t, J=4.9, 4H), 6.54 (s, 1H), 6.75-6.78 (dd, J=8.8, 1H), 7.02-7.03 (d, J=2.9, 1H), 7.20 (s, 1H), 7.31-7.39 (d, J=8.8, 1H); ^13^C-NMR (75 MHz, CDCl_3_) δ: 0.0, 0.8, 29.2, 29.3, 36.9, 50.3, 56.8, 80.8, 115.8, 120.2, 121.5, 126.8, 129.6, 134.0, 135.1, 137.2, 140.5, 150.5, 152.9, 155.5.

***tert*-Butyl 4-[1,10,10-trimethyl-5-oxo-2,3,5,10-tetrahydro-1H-benzo(5,6)silino(3,2-f)indol-8-yl]piperazine-1-carboxylate (9)**

To a solution of **8** (162 mg, 0.36 mmol) in 3 mL CH_3_CN was added NH_4_OAc (5.5 mg, 0.070 mmol) at 0℃. The reaction mixture was stirred for 10 min. Then, *N*-bromosuccinimide (NBS) (133 mg, 0.75 mmol) was added to it and the solution was stirred for 30 min at 0℃. The reaction mixture was warmed to room temperature and stirred for 2 h. The mixture was diluted with NaHCO_3_ aq., and extracted with CH_2_Cl_2_. The combined organic layers were washed with brine, dried over Na_2_SO_4_ and evaporated to dryness. The residue was purified by column chromatography (silica gel, AcOEt/*n*-hexane) to afford *tert*-Butyl 4-[1,10,10-trimethyl-5-oxo-2,3,5,10-tetrahydro-1H-benzo(5,6)silino(3,2-f)indol-8-yl]piperazine-1-carboxylate (**9**) (204 mg, 0.33 mmol, 93％).

^1^H-NMR (300 MHz, CDCl_3_) δ: 0.72 (s, 6H), 1.47 (s, 9H), 2.70 (s, 3H), 2.91-2.95 (t, J=11.7, 2H), 3.03-3.05 (t, J=4.9, 4H), 3.28-3.32 (t, J=8.3, 3H), 3.53-3.56 (t, J=4.9, 4H), 6.54 (s, 1H), 6.75-6.78 (dd, J=8.8, 1H), 7.02-7.03 (d, J=2.9, 1H), 7.20 (s, 1H), 7.31-7.39 (d, J=8.8, 1H); ^13^C-NMR (75 MHz, CDCl_3_) δ: 0.0, 0.8, 29.2, 29.3, 36.9, 50.3, 56.8, 80.8, 115.8, 120.2, 121.5, 126.8, 129.6, 134.0, 135.1, 137.2, 140.5, 150.5, 152.9, 155.5; HRMS (ESI^+^) Calcd for [M+H]^+^, 478.2526, Found, 478.2483 (-4.3 mmu).

***tert*-Butyl 4-bromo-3,5-dimethylbenzoate (10)**

Compound **10** was synthesized according to the literature^5^.

**4-{8-[4-(tert-butoxycarbonyl)piperazin-1-yl]-1,10,10-trimethyl-3,10-dihydro-2H-benzo[5,6]silino[3,2-f]indol-1-ium-5-yl}-3,5-dimethylbenzoate (11)**

To a 100 mL well-dried flask flushed with argon, **10** (182 mg, 0.638 mmol) and THF (10 mL) were added. After the solution was cooled to -78℃, *sec*-BuLi (0.64 mL, 0.64 mmol) was added. After the reaction mixture was stirred at the same temperature for 15 min, the resulting solution was transferred via cannula to a solution of **9** (61 mg, 0.128 mmol) in THF (5 mL). The reaction mixture was stirred for 3 h at room temperature. The reaction mixture was diluted with 2N HCl aq., and extracted with CH_2_Cl_2_. The combined organic layers were dried over Na_2_SO_4_ and evaporated to dryness. The residue was dissolved in trifluoroacetic acid (5 mL) and the solution was stirred at room temperature for 30 min. The reaction mixture was evaporated to dryness and the residue was purified by reversed-phase HPLC (eluent, 24% CH_3_CN/0.1% TFA aq. (0 min) to 64% CH_3_CN/0.1% TFA aq. (20 min); Inertsil ODS-3 (GL Sciences Inc.) 10 × 260 mm; flow rate = 25.0 mL/min) to afford **11** (43 mg, 0.0695 mmol, 52%).

^1^H-NMR (300 MHz, CDCl_3_) δ: 0.45 (s, 6H), 1.49 (s, 9H), 2.91 (s, 3H), 3.03-3.08 (t, J=8.8, 2H), 3.33-3.35 (t, J=4.9, 4H), 3.47-3.51 (t, J=8.3, 2H), 3.60-3.62 (t, J=4.9, 4H), 6.49 (s, 1H), 7.01 (s, 1H), 7.03 (t, J=5.4, 1H), 8.19 (s, 1H), 8.37-8.39 (d, J=8.03, 1H); ^13^C-NMR (75 MHz, CDCl_3_) δ: 27.2, 52.9, 53.2, 58.4, 112.8, 120.0, 120.1, 129.5, 129.7, 130.8, 132.7, 134.5, 134.6, 152.9, 155.8, 182.1; HRMS (ESI^+^) Calcd for [M]^+^, 510.2577, Found, 510.2557 (-2.0 mmu).

**Sodium 5-formylbenzene-1,3-disulfonate (12)**

Compound **12** was synthesized according to the literature^6^.

**SiRpH5.5**

To a solution of **11** (22.1 mg, 43.3 mmol) in MeOH (5 mL) were added **12** (199.8 mg, 64.3 mmol) at room temperature. The reaction mixture was stirred for 30 min at the same temperature, then 2-picoline borane (4.4 mg, 41 µmol) was added. The mixture was stirred for 16 h at room temperature and the purification by reversed-phase HPLC (eluent, 24% CH_3_CN/0.1% TEAA aq. (0 min) to 64% CH_3_CN/0.1% TEAA aq. (20 min); Inertsil ODS-3 (GL Sciences Inc.) 10 × 260 mm; flow rate = 25.0 mL/min) was performed to afford **SiRpH5.5** (8.3 mg, 0.011 mmol, 25%).

^1^H-NMR (400 MHz, CD_3_OD) δ: 0.56 (s, 6H), 2.01 (s, 6H), 2.62 (s, 3H), 2.84 (s, 4H), 3.67 (s, 2H), 3.69 (s, 4H), 3.92 (t, J = 7.3 Hz, 2H), 6.70 (s, 1H), 6.82 (d, J = 9.1 Hz, 1H), 6.91 (d, J = 9.6 Hz, 1H), 7.30 (s, 1H), 7.42 (d, J = 2.7 Hz, 1H), 7.82 (s, 2H), 7.89 (s, 2H), 7.96 (s, 1H), 8.24 (s, 1H); HRMS (ESI^-^) Calcd for [M-2H]^-^, 758.2026, Found, 758.1995 (-3.1 mmu).

The HPLC chromatogram after purification was as follows. The elution was done with a 30 min linear gradient from 0% CH_3_CN/0.1% TFA aq. to 100% CH_3_CN/0.1% TFA aq. (flow rate= 1.0 mL/min); Inertsil ODS-3 (GL Sciences Inc.) 4.6 × 250 mm; Absorbance at 600 nm was detected.

**SiRpH5.5-Dex**

**SiRpH5.5-Dex** was prepared with **SiRpH5.5** by referring to the **SiRpH5-Dex**^5^.

1. **Supplemental References**

1. Miura, Y. *et al.* Identification and quantification of plasma calciprotein particles with distinct physical properties in patients with chronic kidney disease. *Sci. Rep.* **8**, 1–16 (2018).

2. Kabeya, Y. *et al.* LC3, a mammalian homologue of yeast Apg8p, is localized in autophagosome membranes after processing. *EMBO J.* **19**, 5720–5728 (2000).

3. Kimura, S., Noda, T. & Yoshimori, T. Dissection of the autophagosome maturation process by a novel reporter protein, tandem fluorescent-tagged LC3. *Autophagy* **3**, 452–460 (2007).

4. Itoh, M., Yonemura, S., Nagafuchi, A., Tsukita, S. & Tsukita, S. A 220-kD undercoat-constitutive protein: its specific localization at cadherin-based cell-cell adhesion sites. *J. Cell Biol.* **115**, 1449–1462 (1991).

5. Takahashi, S. *et al.* Development of a Series of Practical Fluorescent Chemical Tools to Measure pH Values in Living Samples. *J. Am. Chem. Soc.* **140**, 5925–5933 (2018).

6. Cindric, M., Hamersak, Z. & Dodig, I. Method of detection of amino acid sequence and/or identification of peptides and proteins, by use of a new derivatization reagent and synthesis of 5-formyl-benzene-1, 3-disulphonic acid as derivatization reagent. (2017).

1. **Appendix** Photophysical properties of the pH probes

**
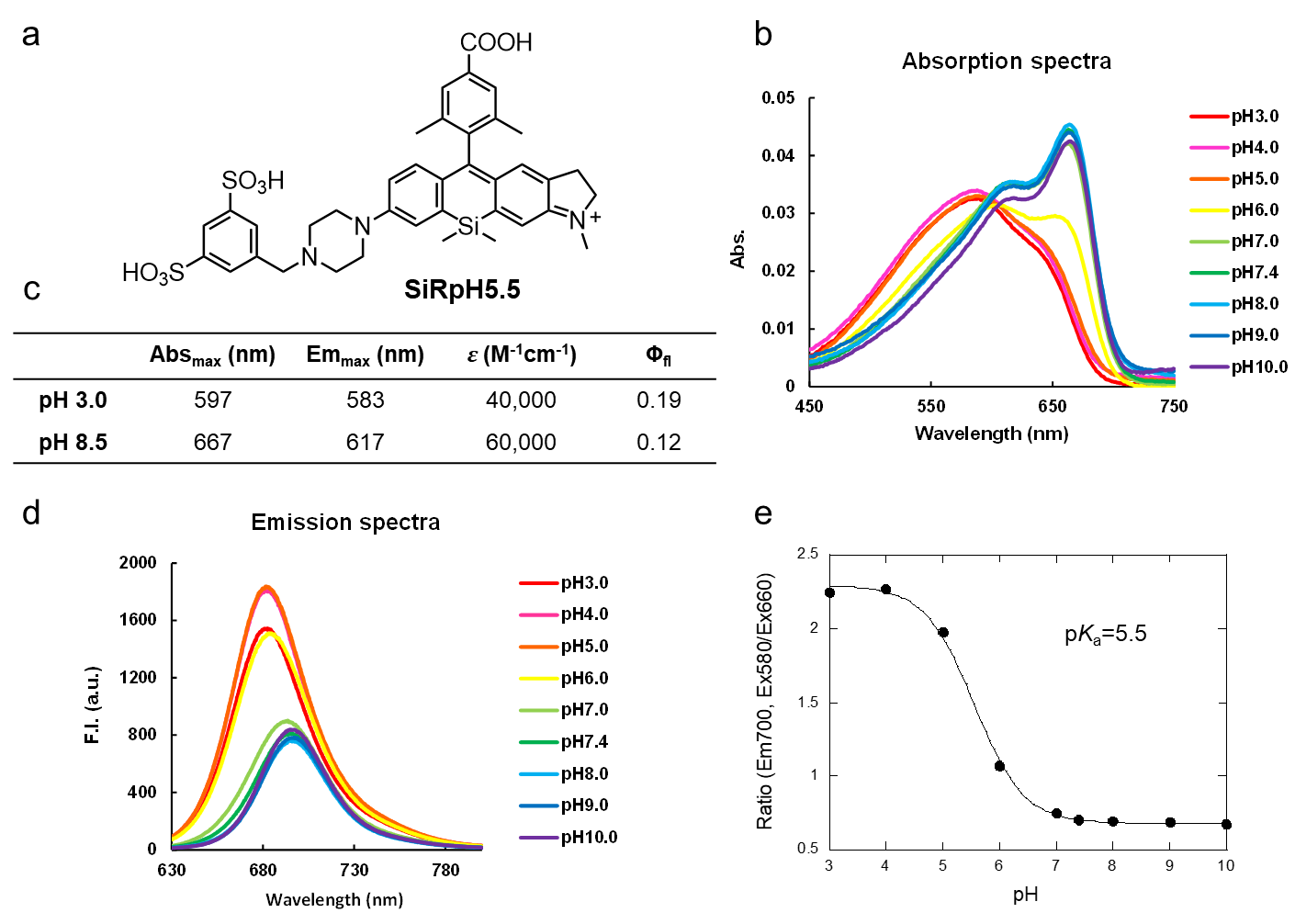
**

**Appendix Fig. 1: Photophysical properties of SiRpH5.5 at various pH values in 100 mM NaPi buffer. a,** Structure of **SiRpH5.5**. **b,** Absorption spectra of 2 µM **SiRpH5.5** at various pH in 100 mM sodium phosphate buffer containing 1% DMSO as a cosolvent. **c,** The photophysical properties of **SiRpH5.5** at pH 3.0 and 8.5. The determination of fluorescence quantum yields were conducted with Hamamatsu Photonics Quantaurus-QY. **d,** Fluorescence spectra (Ex = 610 nm) of 2 µM **SiRpH5.5** at various pH values in 100 mM sodium phosphate buffer containing 1 % DMSO as a cosolvent. **e,** Ratio of the fluorescence intensities at 700 nm excited at 580 nm and 660 nm is plotted against pH. The p*K*_a_ value was 5.5.

**
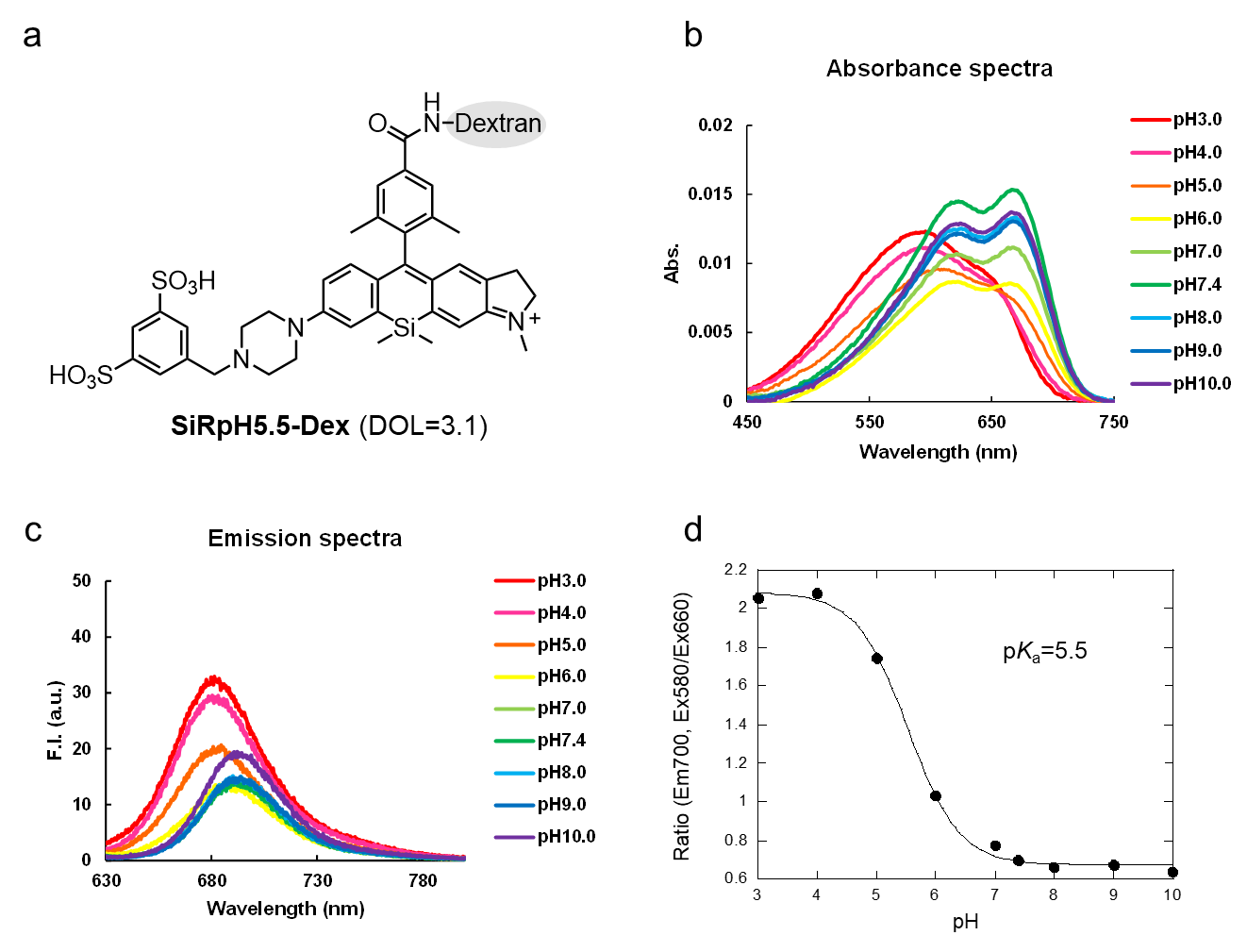
**

**Appendix Fig. 2: Photophysical properties of SiRpH5.5-Dex at various pH values in 100 mM NaPi buffer**. **a,** Structure of **SiRpH5.5-Dex**. **b,** Absorption spectra of 0.31 μM **SiRpH5.5-Dex** at various pH values in 100 mM sodium phosphate buffer. **c,** Fluorescence spectra (Ex = 610 nm) of 0.31 μM **SiRpH5.5-Dex** at various pH values in 100 mM sodium phosphate buffer. **d,** Ratio of the fluorescence intensities at 700 nm excited at 580 nm and 660 nm is plotted against pH.
